# Biclique extension as an effective approach to identify missing links in metabolic compound–protein interaction networks

**DOI:** 10.1093/bioadv/vbac001

**Published:** 2022-01-12

**Authors:** Sandra Thieme, Dirk Walther

**Affiliations:** Max Planck Institute of Molecular Plant Physiology, Potsdam 14476, Germany

## Abstract

**Motivation:**

Metabolic networks are complex systems of chemical reactions proceeding via physical interactions between metabolites and proteins. We aimed to predict previously unknown compound–protein interactions (CPI) in metabolic networks by applying biclique extension, a network-structure-based prediction method.

**Results:**

We developed a workflow, named BiPredict, to predict CPIs based on biclique extension and applied it to *Escherichia coli* and human using their respective known CPI networks as input. Depending on the chosen biclique size and using a STITCH-derived *E.coli* CPI network as input, a sensitivity of 39% and an associated precision of 59% was reached. For the larger human STITCH network, a sensitivity of 78% with a false-positive rate of <5% and precision of 75% was obtained. High performance was also achieved when using KEGG metabolic-reaction networks as input. Prediction performance significantly exceeded that of randomized controls and compared favorably to state-of-the-art deep-learning methods. Regarding metabolic process involvement, TCA-cycle and ribosomal processes were found enriched among predicted interactions. BiPredict can be used for network curation, may help increase the efficiency of experimental testing of CPIs, and can readily be applied to other species.

**Availability and implementation:**

BiPredict and related datasets are available at https://github.com/SandraThieme/BiPredict.

**Supplementary information:**

Supplementary data are available at *Bioinformatics Advances* online.

## 1 Introduction

Identifying novel compound–protein interactions (CPIs) is a central research objective of molecular biology as it can be considered critical for understanding biological systems at the molecular level. Recently published studies reported large-scale experimental screens for the identification of novel interactions between compounds and proteins (Diether *et al.*, 2019; Piazza *et al.*, 2018). In these studies, a large number of potential interactions were experimentally tested with only a relatively small fraction of candidate interactions being validated [around 5% for Piazza *et al.* (2018)]. Thus, augmenting experimental approaches with bioinformatic workflows may help to narrow down the set of candidates for experimental testing, and, thus, increase the rate of successfully validated interactions, while also saving time and resources. This study aims to test the utility of computational approaches that employ the so-called biclique-extension method to serve this goal.

**Fig. 1.**
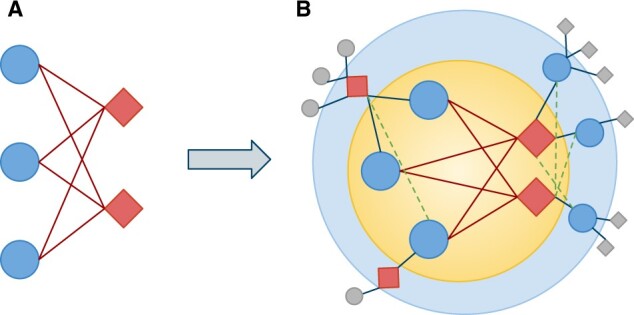
Biclique definition and extension. Bicliques consist of two types of nodes and edges connecting each node of different types (**A**). Here, blue circles represent compounds, *c*, and red squares represent proteins, *p*, while edges represent interactions. The biclique expansion starts with an existing biclique (**B**, yellow inner circle), here consisting of three compounds (*c* = 3) and two proteins (*p* = 2). Next, all compounds and proteins that are directly connected to any member of the biclique are identified. They represent interaction candidates (B, light blue outer circle). Other compounds and proteins of the network that are not directly connected to any member of an existing biclique are not considered (B, gray squares and circles). Finally, interactions are predicted to exist if interaction candidates lack only one edge to become a member of an existing biclique (B, green dashed lines)

CPI networks can be represented as bipartite graphs, in which nodes represent compounds and proteins as the dichotomous entities or groups, and edges represent the interactions between them, but not between compounds or proteins themselves. In a bipartite network, subsets of nodes with the maximum number of possible connecting edges between them actually established are defined as bicliques. Thus, a biclique represents the densest possible connection between a subset of nodes in such a network. We aimed to use bicliques to identify such very closely related sets of compounds and proteins in known CPI networks and to search for interaction candidates in the directly connected neighborhood of these bicliques. The concept is based on the logic that interactions between compounds and proteins are likely true, if, by postulating them, an existing biclique is expanded (see Fig. 1 for a schematic illustration and further explanation of the underlying logic). Biclique-based approaches have been applied to drug-target interaction (DTI) networks for the prediction of novel pharmaceutically relevant compounds or novel target proteins (Daminelli *et al.*, 2012), and for the prediction of protein–protein interactions (Schweiger *et al.*, 2011). Other recently published methods include neural networks based on chemical properties and structure information (Eslami Manoochehri and Nourani, 2020; Tsubaki *et al.*, 2019) as well as random forests based on GO-terms and KEGG pathway enrichment in combination with chemical substructure information (Chen *et al.*, 2016). Also bipartite local models (BLM) have been widely used for DTI predictions, e.g. based on chemical and protein sequence similarity (Bleakley and Yamanishi, 2009; Daminelli *et al.*, 2015) or based on expression data in combination with localization information of enzymes and phylogenetic profiles (Bleakley *et al.*, 2007). BLMs apply network-theoretic approaches for the prediction of interactions that employ local topological information (Cannistraci *et al.*, 2013) to bipartite networks. For recent reviews on DTI predictions, see Lotfi Shahreza *et al.* (2018), Wu *et al.* (2018) and Lim *et al.* (2021).

We aimed to transfer the biclique-extension approach from the field of DTI networks to the identification of as of yet unidentified CPIs in naturally occurring metabolic and cellular networks. Most computational approaches that have been developed to predict CPIs aim at drug–protein interactions. However, metabolite–protein interactions have their own specifics. They evolved naturally and under different selection criteria than drug–protein interactions that may shape binding specificities and strengths differently. Furthermore, while drugs have been designed to bind as single moieties, different metabolites typically bind jointly to the catalytic site of the respective protein enzyme in order for the respective biochemical conversion to proceed, except for isomerase reactions, in which only one substrate binds, or when binding as an allosteric effector. Even in the case of hydrolase reaction, a second molecule (water) has to ‘bind’. Thus, it remains to be explored, how well network-based approaches to predict interactions work when focusing on natural metabolite–protein interaction networks.

The increasing number of experimentally verified interactions in public databases now allows us to search for new interactions solely on the basis of known interactions, without adding any other sources of information to our network. By focusing only on the network structure in our approach, we aimed to develop an efficient network curation method that identifies ‘missing’ interactions and which can augment other, well established computational methods for the prediction of CPIs that follow a more direct molecular-descriptor-based approach (Lim *et al.*, 2021) and to furthermore focus on metabolite-protein, and thus, naturally occurring interactions.

A crucial element for assessing the accuracy of CPI predictions is the availability of known negative interactions, which refers to CPIs that are confirmed to not interact appreciably under natural conditions (Liu *et al.*, 2015). The validation of newly predicted interactions benefits from having both true-positive (TP) and true-negative interactions determined in wet-lab experiments, which are not yet part of the input interaction network the predictions are based on. In contrast to most published CPI prediction methods, we did not only use randomly generated negative samples for the verification of our predicted interactions, as it is difficult to assess for such random data how many positive interactions are actually contained in such random connections. Instead, we used negative interactions as reported experimentally or predicted computationally at high confidence levels.

We analyzed an *Escherichiacoli* CPI network to test the prediction performance on recently published experimental data and also applied the biclique-extension method to a human CPI network for which a computed validation dataset based on a large number of biological features was available. In addition to biclique-based predictions, we studied the network properties, which proved relevant for correct biclique predictions.

## 2 Methods

### 2.1 Overview

**Fig. 2.**
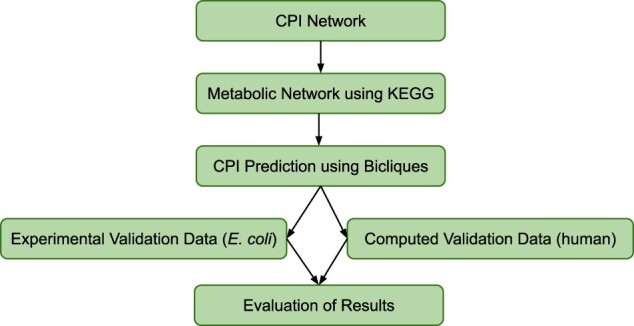
Biclique prediction workflow. Using a CPI network (e.g. STITCH) as input and removing all non-naturally occurring interactions based on information from KEGG, we generated a naturally occurring metabolic network. We identified interaction candidates using bicliques and evaluated the predictions using different validation datasets derived from sampling, experimental and computational studies

We established the following biclique-extension workflow, summarized in Figure 2. First, a reference network of validated interactions was constructed. Here, we computed a CPI network based on data from the STITCH database for *E.coli* and human. Next, we used annotation information from the KEGG database to remove all interactions of drugs from the network to obtain a naturally occurring metabolic/cellular network. We predicted novel interactions based on interaction candidates, which were connected to existing bicliques in the reference network (Fig. 1). For validation of our predictions, two additional datasets were needed. First, a true-positive (TP) dataset of true interactions, which are not part of the reference network and, secondly, a negative dataset for which no interactions could be shown to serve as a true-negative set. Here, we used datasets generated based on experimental data for the *E.coli* network (Piazza *et al.*, 2018) and computationally predicted data for the human network (Liu *et al.*, 2015) to evaluate our results.

### 2.2 The reference network

Information about known and predicted interactions between compounds and proteins of *E.coli* K12 MG1655 and human was downloaded from the STITCH database, used as the primary source of reference CPI network input information (http://stitch.embl.de, version 5.0) (Kuhn *et al.*, 2008; Szklarczyk *et al.*, 2016), including links to other databases, names and SMILES strings of compounds. Protein sequences and links to other protein databases were downloaded from the STRING database (Szklarczyk *et al.*, 2019), UniProt (The UniProt Consortium, 2017), and from LINKDB (Fujibuchi *et al.*, 1998). STITCH database entries of compounds were rendered non-redundant by merging identifiers capturing isoform and salt variants. STITCH also provides a confidence score for every reported interaction, ranging between zero and one and with larger values corresponding to higher confidence (qualitative intervals: low scores of 0.0–0.4, medium 0.4–0.7, high: 0.7–0.9 and very high confidence: 0.9–1).

Out of the total of 1 821 709 reported interactions for *E.coli*, 242 125 interactions were assigned a confidence of ‘medium’ or better. To infer the CPI network, only experimentally verified interactions were included, representing edges in the network (176 100 STITCH interactions). Both, direct as well as transferred confidence scores, i.e. scores assigned by homology from other species, were taken into account. We applied a ‘medium’ experimental confidence score of 0.4 to the STITCH network as a lower threshold, which included 99 487 out of 1 821 709 reported interactions. To test for robustness of our predictions, networks based on confidence score thresholds ranging between 0.4 and 0.6 were tested as well. Thus, we tested our predictions on three networks ranging in size from 37 655 (score > 0.6) interactions to up to 99 487 (score > 0.4) interactions.

Out of the total of 15 473 939 reported interactions in human, 1 545 933 interactions were assigned a STITCH confidence score of ‘medium’ or better and 8 842 952 STITCH interactions carry experimental support. We applied a ‘medium’ experimental (considering direct and transferred) confidence score of 0.4 as a lower threshold on the human network, which resulted in 1 026 207 interactions. A network based on a confidence score of 0.5, including 641 457 interactions was tested as well.

#### 2.2.1 BindingDB input network

A second CPI network, based on the BindingDB (BDB) database (Gilson *et al.*, 2016), was used as input to allow for comparative analyses. Here, all interactions assigned to human and carrying a KEGG compound ID (‘C’ number) were included. This resulted in a network of 11 253 interactions between 2134 compounds and 1299 proteins.

#### 2.2.2 KEGG metabolic pathway network

As a third source of CPI information, KEGG metabolic pathway networks were used as input for biclique extension. KGML files of all pathways reported for *E.coli* and human were downloaded using the KEGG API (i.e. a REST-style Application Programming Interface to the KEGG database resource). Based on this information, enzymes were mapped to reactions and a bipartite network, which connects enzymes and compounds was created. KEGG ‘C’ compound numbers and gene IDs were mapped to ensemble (STITCH) IDs, for validation and comparison of results.

### 2.3 Network construction

Bipartite CPI networks were computed using the R-package igraph (Csardi and Nepusz, 2006; R Core Team, 2016). Nodes of the network represent compounds and proteins. Edges were inserted connecting compounds and proteins, for which known interactions based on the filter criteria described above were reported.

### 2.4 Data cleanup

To exclude unspecific interactions, small compounds, such as ions, were removed from the network. Correspondingly, all interactions with compounds of less than five heavy atoms, as determined from their SMILES strings, were removed from the initial dataset, as done similarly by Daminelli *et al.* (2012). Also, the STITCH and the BDB CPI network contain many interactions of compounds, which are not naturally occurring in the metabolic or cellular network of the corresponding organism, such as antibiotics. We used additional annotation information from KEGG (Kanehisa *et al.*, 2017) to confine our sets of compounds to metabolites and naturally occurring cellular compounds (‘C’ number KEGG compounds). In addition, compounds marked as ‘antibiotics’, and (in *E.coli*) ‘hormones and transmitters’ or ‘steroids’ in KEGG were removed from the network. Also compounds with a drug ID in KEGG (‘D’ number) and without an additional ‘C’ number in KEGG were also removed from the network. Compounds assigned a ‘D’ number, but also a ‘C’ number were retained.

In addition, prior to the biclique calculations, we excluded all interactions with compounds and proteins occurring only once (i.e. one reported interaction only) from the STITCH input network, because such interactions could not be part of any biclique, which we required to consist of at least two compounds and proteins (i.e. each biclique member must have at least two interactions). This reduced the *E.coli* network to 6353 interactions and the human network to a size of 42 158 interactions. This performance step reduced the computational time for the large human STITCH network, and was for consistency and comparability reasons also applied to the STITCH *E.coli* network. For the smaller BDB and KEGG networks, this step was not applied.

### 2.5 Validation data

The prediction performance of the biclique-extension method was tested on two reference CPI networks from two species: *E.coli* and human. For both species, true-positive (TP) interactions were taken as random samples from high-confidence STITCH interactions.

True-negative interaction compound–protein pairs were taken from two different resources. For *E.coli*, we used interactions, which were neither reported in STITCH nor detected interacting in a recent experimental assay in which the interactions were tested (Diether *et al.*, 2019; Piazza *et al.*, 2018).

For human, a true-negative set was taken from a study that aimed to computationally assemble a high-confidence negative CPI-set (Liu *et al.*, 2015).

In both cases, only those validation-set CPIs were considered, for which the corresponding compounds and proteins were found present in the STITCH reference network. The specifics of the validation sets and runs are outlined below.

#### 2.5.1 Validation procedure and data, *E.coli*

As a positive validation dataset, in each of the ten performed prediction runs, 5% (KEGG) or 10% (STITCH) of the true interactions as reported by the respective reference network were randomly chosen and considered predictable TPs (hereafter referred to as positives). All positives were removed from the reference CPI network prior to the biclique detection and subsequent prediction. They were used to calculate the true-positive rate (TPR) of predicted interactions.

A validation set of negative interactions was compiled from experimental data. Piazza *et al.* experimentally tested 34 186 interactions between 20 central compounds (e.g. ATP, ADP, NADP; Supplementary Table S1) and 2525 proteins of interest and reported 1719 interactions. A negative validation dataset for our study was created based on the 32 467 interactions, which were tested by Piazza *et al.*, and reported to not interact in their experiments. A subset of 26 724 interactions consisted of compounds and proteins that were also part of the STITCH network and not known to interact (hereafter referred to as Piazza.negatives). This dataset was expanded by a second, recently published interaction dataset that also reported experimentally tested interactions in *E.coli* (Diether *et al.*, 2019). From the experimental data of Diether *et al.*, an additional set of 1354 tested, but reported as non-interacting compound–protein pairs comprising 55 compounds and 29 proteins was included as true negatives in our study. However, 584 of these supposedly negative interactions were included in the STITCH database of known and predicted interactions. These interactions were removed from the validation dataset of negatives, which finally comprised 863 non-interacting compound–protein pairs (hereafter referred to as Diether.negatives). By adding this dataset, the number of compounds included in the validation data (negatives) was increased from 20 to 57. As 172 interactions of Diether.negatives were already contained in the Piazza.negatives, the combined negatives list finally included 27 415 unique experimentally verified non-interactions between 57 chemicals and 2474 proteins.

Depending on the chosen confidence score of the reference STITCH network, 10 720 of these negative interactions (negatives) were available for prediction; i.e. both the compounds and proteins were present in STITCH. They were used to calculate the false-positive rate (FPR) of predicted interactions. Also, more than 8000 of negatives were available for evaluation of prediction results using the KEGG database.

Only predictable interactions were taken into account to calculate true-positive and false-positive interactions and rates, i.e. only interactions between compounds and proteins, which were both included in the reference network, and, thus, could be predicted by biclique extension.

#### 2.5.2 Validation procedure and data, human

A positive validation dataset was created by randomly choosing 5% of interactions from the input network.

A dataset of negative interactions was taken from Liu *et al.* (2015). This study published highly reliable negative interaction sets combining various chemical, structural and interaction information. From the provided negatives dataset, 39 758 out of 40 381 compounds and 1974 out of 2027 proteins could be successfully mapped to STITCH IDs, yielding a total of 369 276 negative interactions. Of these, 15 865 were predictable negative interactions (negatives) with chosen confidence score of the reference STITCH network of 0.4. Using the KEGG input network, ∼4000 negatives, and for the BDB network >15 000 negatives, were available for validation. All interactions between compounds and proteins that were also included in the input network were considered to be predictable interactions.

### 2.6 Biclique calculation and extension

All maximum bicliques of compounds and proteins in the CPI network were calculated using the R-package biclique (Lu *et al.*, 2020; Zhang *et al.*, 2014). Using maximum-size bicliques makes sure that no bicliques of a certain size that are fully contained in larger bicliques are considered separately. However, overlapping bicliques are possible, and therefore, edges, representing interactions between two nodes, can be members of multiple bicliques.

A minimum number of two and up to nine nodes on either side of the biclique was tested, representing the minimum number of compounds and proteins of each biclique. Thus, the smallest considered bicliques consisted of two proteins and two compounds.

Candidates for novel interactions between compounds and proteins in the network were searched in the directly connected neighborhood of existing biclique-member nodes, i.e. proteins and compounds, which are connected to at least one node of the biclique. Only compounds and proteins, which become part of an existing biclique by insertion of exactly one connecting edge, were considered as novel interaction candidates (see Fig. 1 for a schematic illustration). In addition, an insertion of two edges was tested for bicliques with more than four nodes on the corresponding side.

### 2.7 Molecular similarity measures

The similarity of compounds was estimated based on the Tanimoto index with structural features derived from the SMILES string and using the R-package RxnSim (Giri *et al.*, 2015). The similarity of proteins was assessed based on pairwise protein-based BLAST alignment scores. *E.coli* protein sequences were downloaded from UniProt, ID: UP000000625, strain K12. Given the set of proteins included in the *E.coli* dataset, an all-against-all blastp search was performed with the *E*-value threshold set to 10 to allow for weak alignments to be reported and considering one (the best) high-scoring pair per sequence pair only. Otherwise, default blastp settings were used. Cohen’s-d effect sizes were calculated using the R-package effsize (Torchiano, 2016).

### 2.8 Randomization

To investigate the importance of the underlying reference network of experimentally verified interactions for predictions, we created a randomized network altering the reference interaction network. To generate a random bipartite network, the R-package BiRewire (Gobbi *et al.*, 2020) was used. BiRewire uses the edge switch algorithm to preserve node degrees of the input network. This randomized network was also used for biclique calculation and extension.

### 2.9 Prediction performance metrics

Prediction results were assessed with regard to TPR (or sensitivity or recall), FPR, and F1-score, and precision as commonly defined.

(1) $$\mathrm{TPR}=\frac{\mathrm{TP}}{P};\mathrm{FPR}=\frac{\mathrm{FP}}{N};\;F1=\frac{2TP}{2\;\mathrm{TP}+\mathrm{FP}+\mathrm{FN}},\;\mathrm{Precision}=\frac{\mathrm{TP}}{\mathrm{TP}+\mathrm{FP}},$$

where *P* is the number of positives, *N*—the number of true negatives (no interaction), TP—true-positive predictions, FP—false-positive predictions and FN—false-negative predictions, assessed based on the positive and negative validation datasets described above.

### 2.10 KEGG enrichment analysis

To inspect the obtained prediction results in terms of biological function and their biochemical processes, we performed a KEGG enrichment analysis on the *E.coli* network using the R-package clusterProfiler (Wu *et al.*, 2021; Yu *et al.*, 2012).

### 2.11 Comparison with other methods, DeepConv-DTI

We compared our performance rates to those obtained by a recently published deep-learning method DeepConv-DTI (Lee *et al.*, 2019), which itself was evaluated against several state-of-the-art approaches to allow even broader comparisons. We obtained the source code from GitHub (https://github.com/GIST-CSBL/DeepConv-DTI) and used the rcdk package to obtain Morgan fingerprints from SMILES strings (Guha, 2007). For a comparison with DeepConv-DTI, we generated a training dataset to train the model with our input data, as well as a test dataset for prediction and validation. For training, we used the same human STITCH input network for both methods. This input network was reduced from 44 322 interactions to 36 114 interactions, since SMILES strings and protein sequences were not available for all compounds and proteins in the STITCH network. As positives for the test data, we sampled 5% (*n* = 1671) of the input STITCH network and removed these interactions from the training dataset. As DeepConv-DTI also needs negative data for training, we added negatives from the dataset we used for validation (Liu *et al.*, 2015), to the input network. We randomly sampled the same number of negative interactions as we had positive interactions (*n* = 34 443), and only used negative interactions in which either the protein or the compound was also included in the positive dataset (one interaction partner). All negative interactions between compounds and proteins, which were both included in the input STITCH network (both interaction partners), were used as negatives for validation of the test data (*n* = 15 096). Thus, the test dataset was identical to the dataset we used to validate our own method. We trained and tested DeepConv-DTI using the same parameters given in the GitHub examples by the authors (using convolution, training for 15 epochs, and using Morgan fingerprint with radius of 2 and size 2048). Default settings of DeepConv-DTI were used (0.5 score threshold for binary classification).

### 2.12 Software and data availability

The developed method, called BiPredict, implemented as an R-script along with relevant data used in this study is available at https://github.com/SandraThieme/BiPredict.

## 3 Results

### 3.1 Properties and structure of the STITCH CPI networks

To apply biclique extension to predict novel CPIs, we first compiled an *E.coli* reference interaction network based on data from the STITCH database. The *E.coli* network included 6894 interactions between 177 compounds and 1906 proteins, and after removing degree-one interactions (as they are not relevant for our method), 6353 interactions between 160 compounds and 1381 proteins (Table 1).

**Table 1.** Number of interactions (*N*_i_), compounds (c) and proteins (*p*), and network density *D* = *N*_i_/(*c* × *p*) in the *E.coli* and human network after different filtering steps

Dataset	*E.coli*	Human
Number of interactions, density STITCH network confidence threshold =0.4	99 487 (*c* = 24 602 *p* = 2562) *D* = 1.58E-3	1 026 207 (*c* = 410 253, *p* = 9047) *D* = 0.28E-3
Number of interactions, density metabolic network after cleanup	6894 (*c* = 177, *p* = 1906) *D* = 2.04E-2	44 322 (*c* = 2115, *p* = 7542) *D* = 0.28E-2
Number of interactions, density without single (degree =1) interactions	6353 (*c* = 160, *p* = 1381) *D* = 2.88E-2	42 158 (*c* = 1598, *p* = 5885) *D* = 0.45E-2

*Note*: As node degree =1 interactions were not considered in the biclique computations (as they cannot contribute to our predictions), we list them separately.

The human network consisted of 44 322/42 158 interactions including 2115/1598 compounds and 7542/5885 proteins, with and without degree-one interactions, respectively (Table 1). The maximum node degree of compounds was 2959 (selenomethionine), the mean degree was 20.9 and the median was 3. The maximum degree of proteins was 100 [5-hydroxytryptamine (serotonin) receptor 2 A], the mean degree was 5.9 and the median 3. Judging by their network density, the *E.coli* network had a much higher network density than the human network (7/6-fold difference, Table 1 with/without degree =1 interactions).

**Fig. 3.**
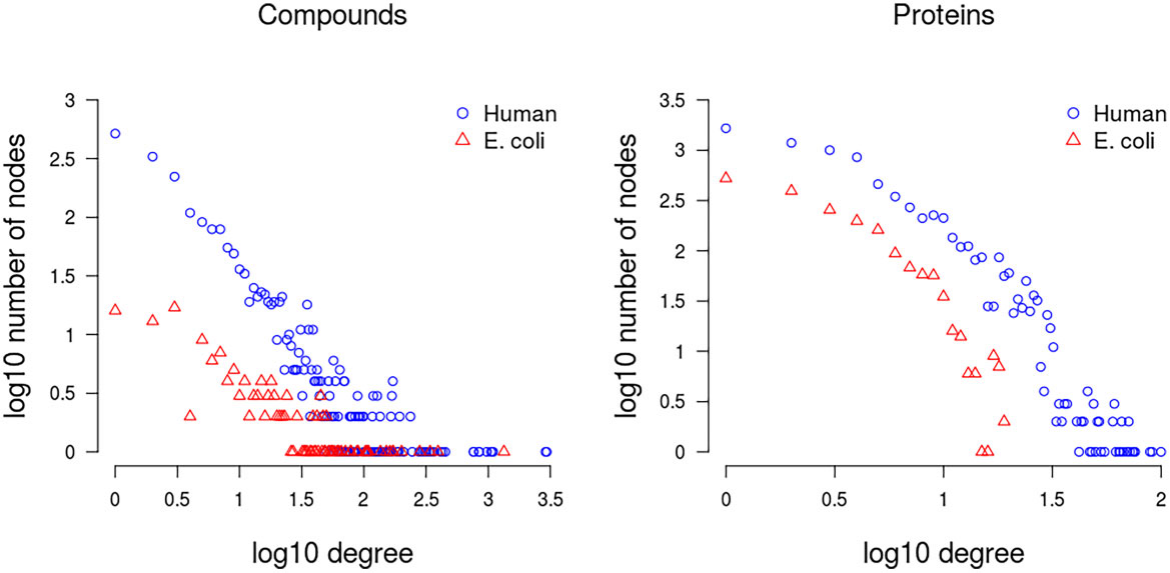
Degree distribution of compounds (left panel), and proteins (right) in the *E.coli* and human STITCH reference network. The *x*-axis represents the node degrees (log10) and the *y*-axis the frequency of nodes having that degree (log10)

Despite their different sizes, with many more interactions reported for human than for *E.coli*, both networks showed similar degree distributions when recorded for compounds and proteins, respectively (Fig. 3). For a compound-centric view, both networks follow a power-law (linear relationship in log–log scale, Fig. 3, left panel), with the human data shifted to higher counts due to its larger network size. Power-law degree distributions have been found to be a characteristic of biological networks (Lima-Mendez and Helden, 2009). By contrast, a power-law was less obvious for a protein-centric degree distribution (Fig. 3, right panel) with counts dropping faster than expected from a power-law alone.

**Fig. 4.**
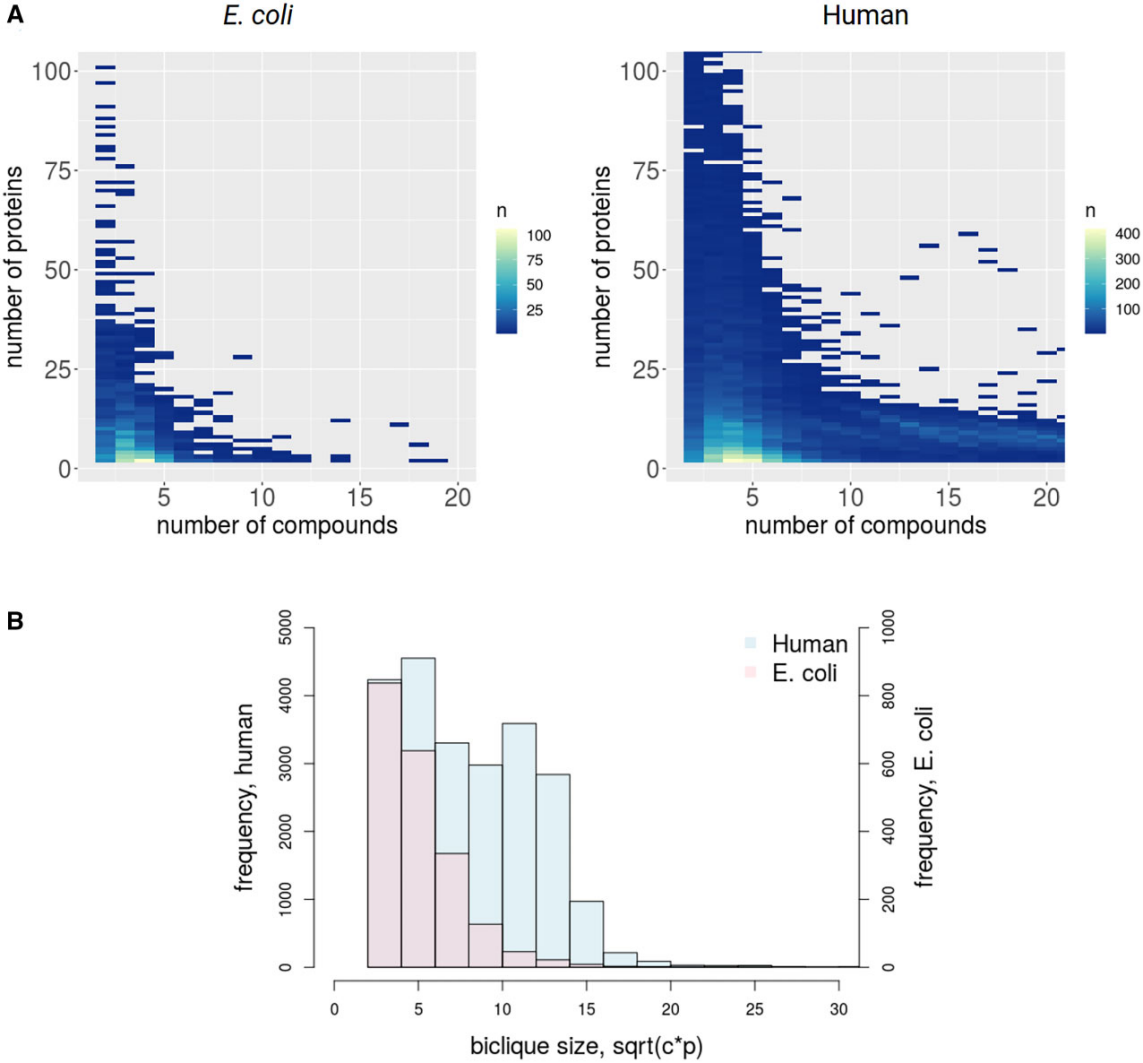
Size-dependent biclique frequency. (**A**) Left: *E.coli* heatmap for all compound/protein biclique sizes up to 100 proteins and 20 compounds. Bicliques including four compounds and two proteins are the most common with *n* = 106 (total number of bicliques *n* = 2022). (A) Right: human heatmap for all *c*/*p*-biclique sizes up to 100 proteins and 20 compounds. Bicliques including four compounds and two proteins are the most common with *n* = 419 (total number of bicliques *n* = 22 879). (B) Comparison of frequencies of bicliques of different sizes captured as a single number to allow for better comparison of *E.coli* versus human and defined as sqrt(*c*×*p*), where *c* is the number of compounds and *p* the number of proteins (histogram clipped at size sqrt(*c×p*)=30)

With regard to bicliques, 2202 bicliques were detected in the *E.coli* network, with small bicliques being most prevalent and with *c*/*p*-biclique size *c* = 4 and *p* = 2 being the most frequent biclique (106 times in the input network). The analyzed human network contained 22 879 bicliques. Here, larger bicliques were more frequent relative to *E.coli* and bicliques of size *c* = 4 and *p* = 2 being most frequent with 419 occurrences (Fig. 4).

#### 3.1.1 Structural similarity of compounds and proteins of the same biclique

**Fig. 5.**
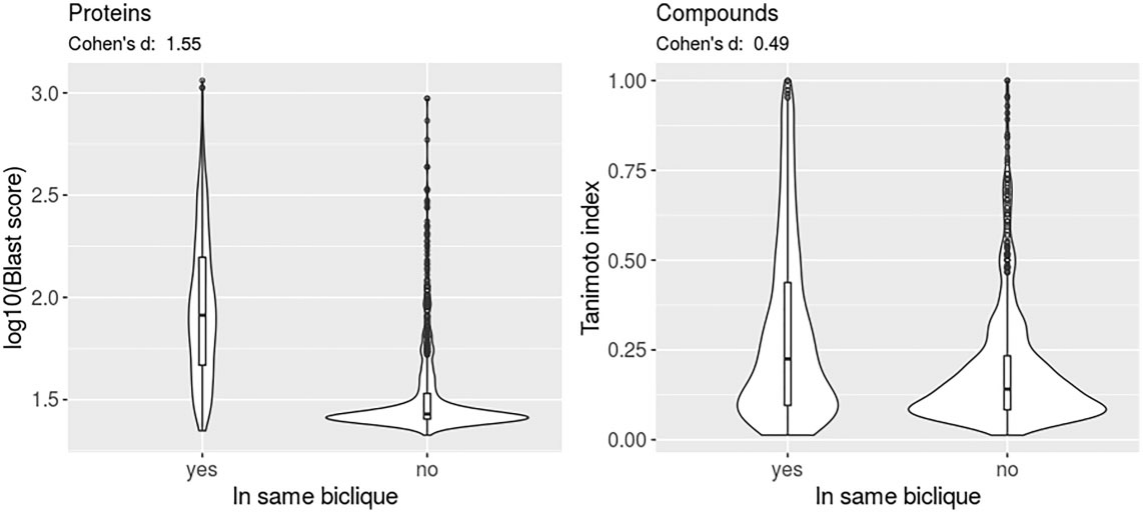
Molecular similarity of compounds and proteins within the same and between different bicliques. Violin plots of molecular similarity measures (blastp-score for proteins, Tanimoto index for compounds, see Section 2) of compounds and proteins that are part of the same biclique or not with Cohen’s *d* effect sizes indicated, respectively. Distributions are based on 1000 randomly selected within and across-different molecule pairs. Corresponding Wilcoxon rank sum test *P*-values: compounds: <2.2E-16, proteins: <2.2E-16

In bicliques, by definition, all member-compounds interact with the same set of proteins, and likewise, all member-proteins interact with the same set of compounds. As this agreement with regard to their respective molecular binding partners must have a molecular basis, it seems reasonable to hypothesize that proteins and compounds that are part of the same biclique are structurally similar. Indeed, proteins belonging to the same biclique show significantly higher sequence similarity (*P* < 2.2E-16, with sequence similarity used as a proxy of structural similarity) than found between proteins that belong to different bicliques (Fig. 5). Likewise, compounds that are members of the same biclique display greater chemical similarity than compounds that are not members of the same bicliques (*P* < 2.2E-16) (Fig. 5). Interestingly, the difference of similarity within or across bicliques seems stronger for proteins (Cohen’s *d* effect size =1.55) than for compounds (Cohen’s *d* = 0.49). Assuming that the difference of similarity scale does not affect effect size, this may reflect that proteins may have several binding sites (e.g. for substrate and co-factors) such that with regard to compounds, diversity is greater than for proteins.

### 3.2 Evaluation of the biclique-extension method

#### 3.2.1 Performance on STITCH CPI networks

Based on the obtained reference networks, we employed the logic of biclique extension as laid out in Figure 1 to predict novel interactions between compounds and proteins.

First, we tested the performance of the biclique-extension method on the *E.coli* CPI reference network. Performed in a cross-validation-type test setting, we calculated TPRs and FPRs to evaluate our prediction results. In each of the 10 performed test runs, randomly chosen 10% of true interactions were considered unknown for the purpose of prediction, allowing to compute mean rates and associated standard deviations on the respective hold-out set.

**Fig. 6.**
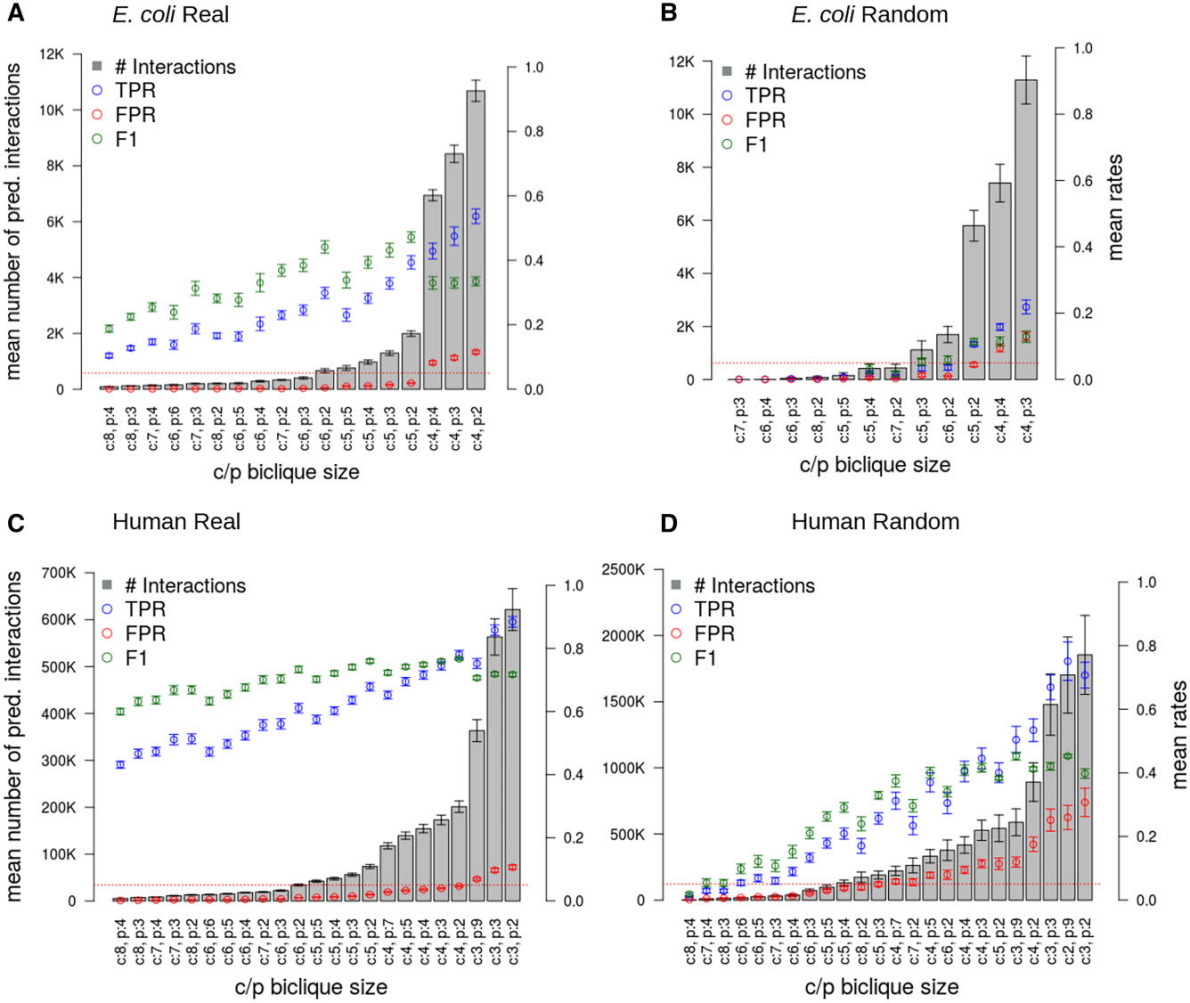
Results of the biclique extension-based interaction predictions using a confidence threshold of 0.4 on the STITCH network and with 10 repetitions on different random samples. Sorting of data corresponding to different biclique sizes in ascending order of the number of predicted interactions (gray bars, average of 10 runs). Left ordinate axis: number of predicted interactions, right ordinate: rates representing fractional values of TPR, FPR, and F1-score. Error bars correspond to standard deviations. The red dotted line marks the 0.05-line to allow for better visual clarity with regard to FPR. Note that the shown *c*/*p*-biclique sizes represent a subset of all possible biclique sizes, which are shown in Supplementary Tables S3 and S4 for *E.coli*, S7 and S8 for human. (**A**) and (**B**) show results obtained for *E.coli*, (**C**) and (**D**) for human. The best obtained biclique size for *E.coli* was *c* = 5 and *p* = 2, with maximal TPR with concurrent FPR<0.05 and highest F1-score

The number of predicted interactions strongly depended on the sizes of bicliques with tested size-thresholds ranging from two to eight for the minimum number of nodes on either side of the biclique [nodes representing compounds (*c*) and proteins (*p*)], from 87 to ∼171 154 interaction candidates (Fig. 6). Note that biclique size refers to biclique-size thresholds, which means that we defined the minimum size of bicliques, which were considered for calculation of performance measures. For example, by applying a threshold of *c* = 5 and *p* = 2, all interactions were predicted using bicliques of this size and, in addition, all occurring bicliques of larger size, with higher or equal number of compounds (*c*) and proteins (*p*), such as *c* = 6 and *p* = 2 were also included.

In general, smaller maximal biclique sizes resulted in more predicted interactions, as the predicted interactions for larger bicliques were also included, and also, due to their increased occurrence in comparison to larger bicliques (Fig. 4). The highest F1-score, in this case also associated with the highest fraction of TP interactions with a FPR below 0.05 out of all predicted interactions, was obtained by using *c*/*p*-biclique-size thresholds of five compounds (*c* = 5) and two proteins (*p* = 2) for the *E.coli* network (Fig. 6A). Applying these *c*/*p*-biclique-size parameters resulted in an average TPR of 0.39 (Fig. 6A and Supplementary Table S3). To test whether the biclique-extension method proves both sensitive and specific and whether it exploits actual biological information as present in the used reference network, we compared the obtained TPRs and FPRs with corresponding rates obtained after randomization of the network using the edge switch algorithm. As expected, for randomized data, we obtained significantly lower TPRs in comparison to real data, e.g. TPR of 0.11 for *c* = 5, *p* = 2 (Fig. 6B and Supplementary Table S4). By contrast, the FPRs observed in real network data and random data were found at similar levels.

As some compounds, in particular those that act as co-factors, such as ATP or NADPH bind to many proteins—those compounds are often dubbed ‘currency metabolites’, we checked how removing them (for a complete list of compounds considered ‘currency metabolites’, see Supplementary Table S14), impacts the prediction performance. While the number of predicted interactions dropped significantly (from 1994 to 457 for biclique size *c* = 5, *p* = 2), which is to be expected, the prediction performance was affected only slightly (F1-scores 0.47 versus 0.40) (Fig. 6A and Supplementary Fig. S5 and Table S15). Thus, the reported prediction performance does not only rely on high-degree interactors.

We also tested the performance when allowing the addition of two edges to declare a compound or protein to be a member of the corresponding biclique. Here, we considered larger bicliques only (minimum number of four compounds or proteins on the corresponding side), to better balance added versus pre-existing interactions. As expected, this resulted in an increased number of predicted interactions for these larger bicliques (Supplementary Fig. S5) as well as increased the FPRs. The prediction performance was comparable with a maximum F1-score of 0.42 and could not be increased compared to allowing only one edge addition.

We also applied the biclique-extension method to the larger human CPI network, which included 44 322 interactions (confidence threshold of 0.4 and after filtering). In ten performed validation runs, we used a set of 5% randomly drawn true interactions of the input network as positive controls, which were removed from the network prior to prediction, and a downloaded set of negative interactions as true negatives (see Section 2). In dependence of the applied thresholds, ranging from two to nine for the minimum number of compounds and proteins on either side of the biclique (maximum *c*/*p*-biclique size), 1648 to ∼1.6 million candidate interactions were predicted for the human network (Fig. 6C and Supplementary Table S7). The TPRs showed an increase with decreasing biclique size, while the FPRs were below 0.05 for all bicliques with more than four compounds and two proteins (Fig. 6C and Supplementary Table S7). The highest F1-score and, thus, the best biclique-size threshold was obtained for *c* = 4, *p* = 2 (F1 = 0.77), followed by *c* = 5, *p* = 2 (F1 = 0.76), which were also found to perform best in *E.coli* (Fig. 6A). The associated TPRs of 0.78 and of 0.68 were much higher compared to the *E.coli* network. We also tested the insertion of two edges for larger bicliques. This showed no major effect on the mean FPRs and the mean TPRs (Supplementary Fig. S4). For a randomized input network, the number of predicted interactions increased significantly compared to the real network (Fig. 6D and C). The *c*/*p*-biclique size *c* = 4, *p* = 2 yielded a mean number of 201 212 predicted interactions in the real network and 983 616 interactions in the randomized network (Supplementary Tables S7 and S8). As expected, TPRs decreased, FPRs increased and F1s decreased accordingly in the randomized network and consistently across all biclique threshold sizes (Fig. 6D and C).

**Fig. 7.**
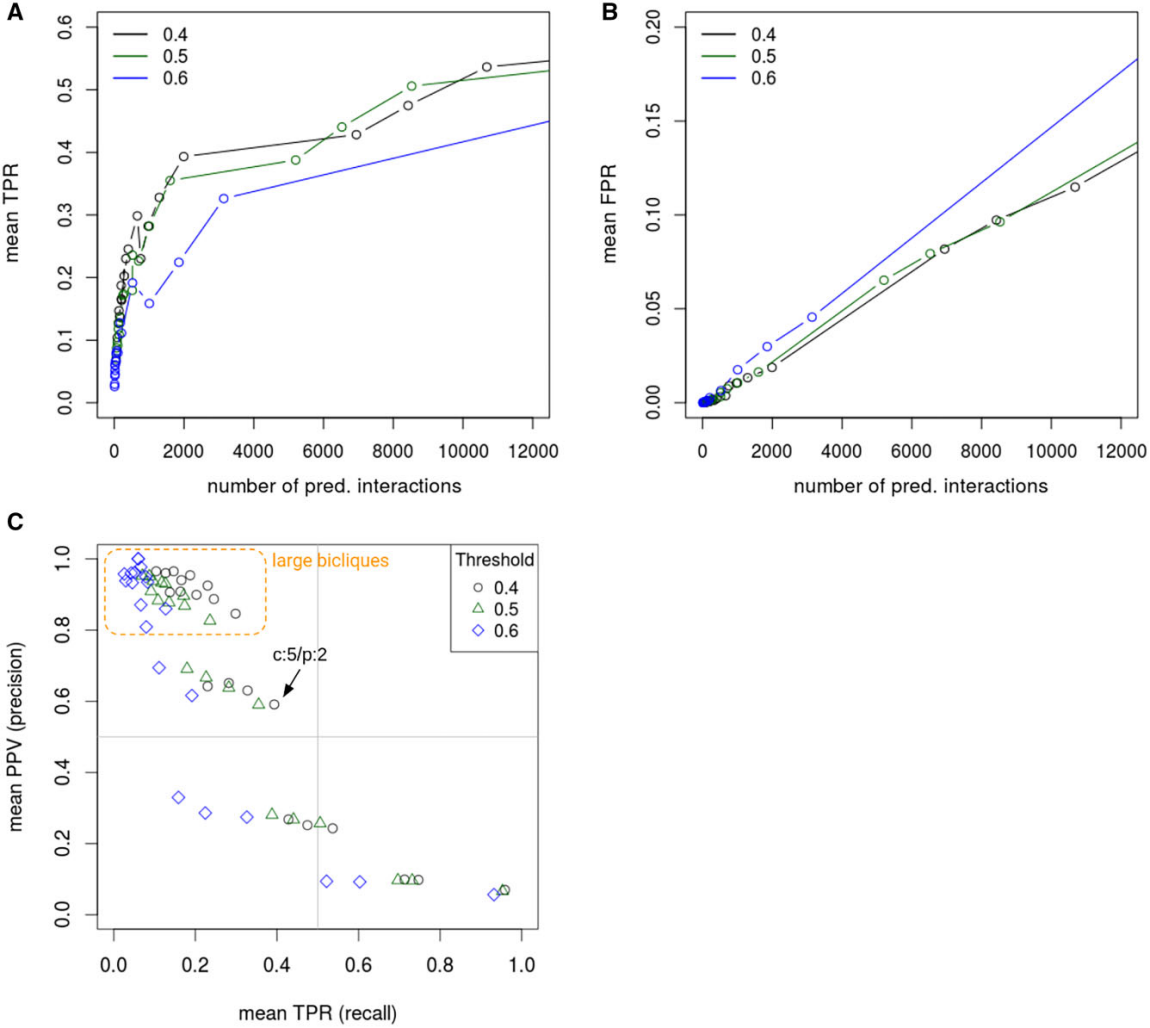
Effect of selected STITCH confidence threshold on TPR, FPR and positive predictive value (PPV). Mean TPRs (A) and mean FPRs (B) in dependence on the number of predicted interactions, and mean TPR in relation to the mean PPV (C) in the *E.coli* interaction network. All performance measures were calculated for prediction results with data from ten repeat runs of different random sample sets. Colors represent the three different confidence thresholds applied to the STITCH network to create the input network. Every circle/point corresponds to a particular *c*/*p*-biclique size as listed in Figure 6. Note the different scales of the *y*-axes in (A) and (B). Note, the effect of many more known negatives (∼9000) than positives (∼600), prevents reaching higher precision. Large bicliques with *c* = 6–8 yielded high precision but low recall. The found optimal (highest F1-score) biclique size (*c*: 5, *p*: 2) is indicated in the graph. There are only slight differences between the PPV and TPR of networks based on different STITCH confidence thresholds, especially for the 0.4 and 0.5 thresholds

As predictions can be expected to critically depend on the validity of the input CPI network, we tested different STITCH confidence thresholds used in the input network construction. In general, we obtained very similar TPRs and FPRs for each tested confidence threshold (Fig. 7), indicating that interactions predicted by the biclique method are not strongly dependent on the underlying levels of confidence in the analyzed interaction network. Among the three tested thresholds, confidence scores 0.5 and 0.4 (Fig. 7A and B, green and black line) yielded the highest TPRs and the lowest FPRs, while the network with highest confidence score 0.6, surprisingly, performed worse, possibly explained by the smaller networks, and thus reduced information in a biclique sense, associated with more stringent thresholds.

To determine optimal parameter settings, we also inspected the precision-recall statistic for the *E.coli* networks based on different STITCH confidence scores and maximum biclique sizes. Consistent with our findings reported in Figure 7A and B, we obtained the best results applying confidence thresholds of 0.4 and 0.5 (Fig. 7C). As expected, larger maximal biclique sizes resulted in higher precision. Here, the biclique-based prediction rests on more support, because a larger number of interactions are known in these bicliques already rendering the logic of biclique extension more applicable. And that logic states that, if a compound binds to all but one *n* other proteins that another set of *m* compounds bind to, then, the one missing interaction for that compound likely occurs as well. And with larger *n* and m, this logic is more compelling and for structural reasons (Fig. 5). The same holds for proteins and their interactions with compounds.

#### 3.2.2 Performance on BDB CPI networks

We also tested the performance of the biclique-extension method on a second human input network to demonstrate the independence from the input network and the reliability of predictions. Since for *E.coli*, BDB contained only 529 interactions with KEGG-‘C’ numbers, we focused on human data only.

The BDB network (Gilson *et al.*, 2016), used as a second CPI input network, contained fewer interactions (*n* = 11 253) than the STITCH network (*n* = 44 322). A total of 1232 compounds and 792 proteins could be mapped to STITCH IDs, which resulted in 7105 comparable interactions. An intersection of 1367 interactions between 466 compounds and 377 proteins was contained in both input networks. This resulted in a set of 174 315 interactions, which were predictable in both networks (maximum possible intersection of predictions). Using the STITCH input network, 24 543 of these interactions, and using the BDB input network, 42 550 interactions were predicted. The obtained overlap of 9024 interactions, predicted using both networks as input, was 1.5 times larger than that predicted by chance (p ≪ 0.01, hypergeometric test). Also, the obtained performance was comparable. Using BDB as input and using a *c*/*p*-biclique size of *c* = 5, *p* = 2 resulted in an F1-score of 0.75, and with *c* = 4, *p* = 2, F1-score =0.75 (Supplementary Fig. S6 and Table S16). The highest F1 using the STITCH input network was obtained for *c* = 4, *p* = 2 (F1 = 0.77), followed by *c* = 5, *p* = 2 (F1 = 0.76). Thus, very similar performances were obtained for two rather different networks used as input.

#### 3.2.3 Performance on KEGG metabolic pathway networks

**Fig. 8.**
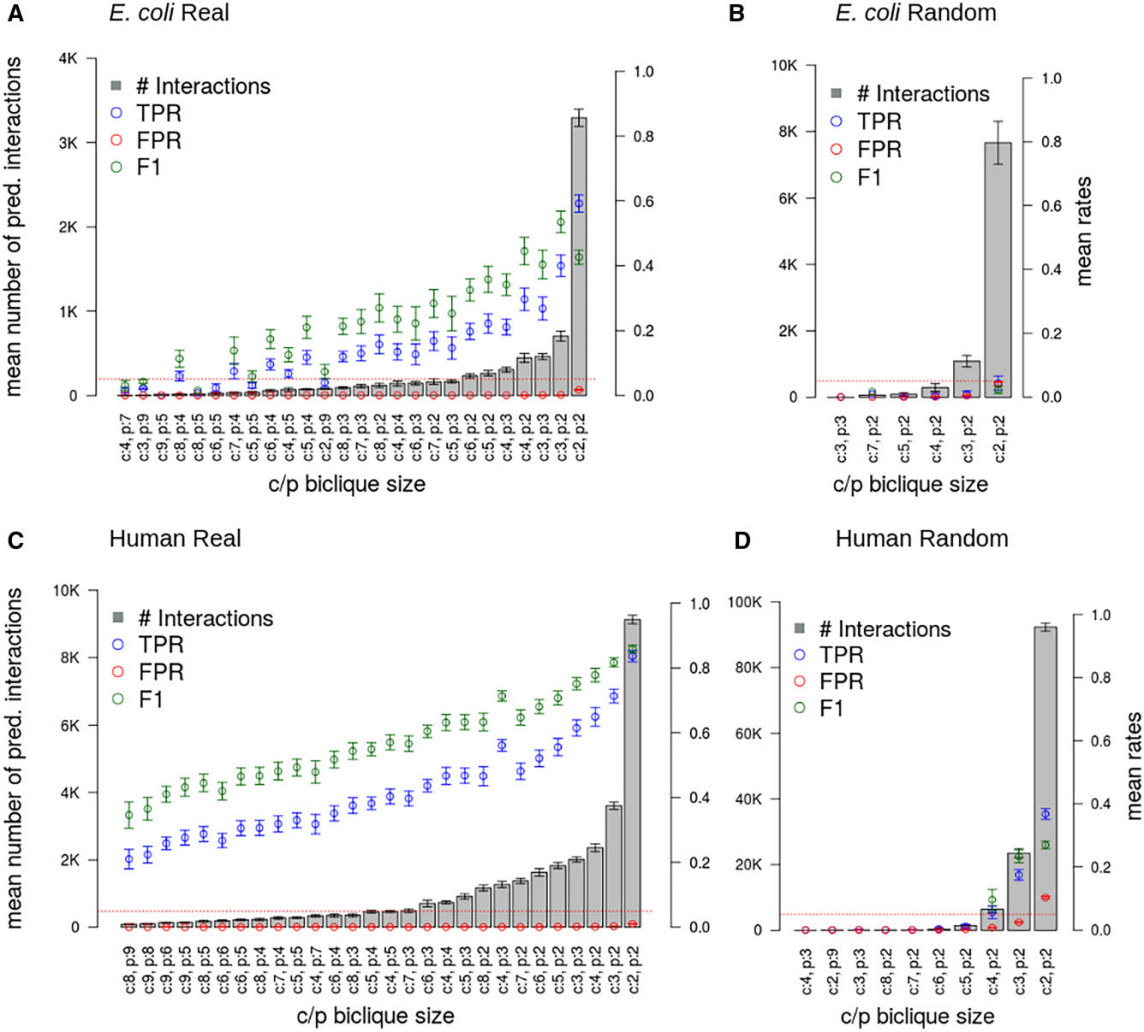
Results of the biclique extension-based interaction predictions for *E.coli* (upper row) and human (lower row) using the KEGG pathway network as input and with ten repetitions on different random samples (5% removed true interactions). On the left-hand side, the results for the real network are shown (A/C) and on the right-hand side the results for the randomized network (B/D). Sorting of data corresponding to different biclique sizes in ascending order of the number of predicted interactions (gray bars, average of ten runs). Left ordinate axis: number of predicted interactions, right ordinate: rates representing fractional values of TPR, FPR, and F1-score. Error bars correspond to standard deviations. The red dotted line marks the 0.05-line to allow for better visual clarity with regard to FPR. See Supplementary Tables S18–S21 for further information

The *E.coli* metabolic pathway network consisting of metabolic reactions between compounds and enzymes, and extracted from KEGG, comprised 3079 unique interactions. For 2930 interactions, the compound ‘C’-numbers could be mapped to STITCH IDs. The network finally included 854 compounds and 894 proteins/enzymes, which corresponds to a network density of 0.38E-2. Using BiPredict and a *c*/*p*-biclique size of *c* = 2 and *p* = 2, 3436 interactions were predicted, with an associated mean TPR of 0.59 and mean FPR < 0.02 (Fig. 8A and Supplementary Table S18). A total of 1621 (∼47%) of all predicted interactions could also be found in the STITCH database with an associated mean/median combined confidence score reported in STITCH for those interactions of 0.62/0.76, respectively, which is significantly higher than that reported for the whole *E.coli* STITCH database (0.28/0.21, Wilcoxon rank sum test *P*-value <2.2E-16). Thus, BiPredict-predicted interactions are of high confidence. Testing different *c*/*p*-biclique sizes, the maximum F1-score of 0.53 was obtained for *c*/*p*-biclique size *c* = 3, *p* = 2, with an associated TPR 0.4, a FPR of 0.001 and a precision of 0.81 (Fig. 8A and Supplementary Table S18). By contrast, the TPRs obtained for the randomized network did not exceed 0.04 with a mean FPR of 0.04, despite the number of predicted interactions being more than doubled compared to the real network (Fig. 8B and Supplementary Table S19).

For human, 7941 unique interactions were obtained from the whole human KEGG-reaction network, of which 6477 interactions between 1120 compounds and 1160 proteins remained after mapping to STITCH IDs. This corresponds to a network density of 0.5E-2. Using BiPredict and *c*/*p*-biclique size of *c* = 2 and *p* = 2, 6929 interactions were predicted, with an associated mean TPR of 0.84 and a mean FPR of 0.01 (Fig. 8C and Supplementary Table S20). As observed for *E.coli*, judging from the set of interactions that are also contained in STITCH [2324 (∼34%) of all predicted interactions], BiPredict predictions are high-confidence interactions (mean/median confidence score 0.60/0.70 relative to the whole human database 0.27/0.22, Wilcoxon rank sum test *P*-value <2.2E-16). The maximum F1-score of 0.86, was also obtained for *c*/*p*-biclique size *c* = 2, *p* = 2, with an associated precision of 0.88 (Fig. 8C and Supplementary Table S20). The largest mean TPR obtained for randomized networks was 0.37 with an associated mean FPR of 0.10 and precision of 0.21 (Fig. 8D and Supplementary Table S21). The number of predicted interactions, for which these performance rates were obtained, was increased 10-fold compared to the real network.

Taken together, also for the metabolic-reaction-based CPIs, biclique extension yields high-confidence CPI predictions.

#### 3.2.4 Biclique extension holds the potential to increase the rate of experimentally validated interactions

We aimed to identify the prediction parameters that would be effective in reducing the number of experimental tests needed to obtain a set of validated novel interactions. Our prediction results for the STITCH *E.coli* network with maximum F1-score, maximum sensitivity (TPR) and associated FPRs below 0.05, resulted in a confirmation rate (i.e. ratio of TP interactions to the number of predicted interactions) of at least 0.12 (Table 2). In comparison to the broad scale experimental studies with confirmation rates of about 0.05 (Piazza *et al.*, 2018), this substantially increases the rate of validated interactions. Relying on larger bicliques would significantly increase the expected confirmation rate (0.71 for *c* = 8, *p* = 4), but at the expense of a much smaller number of predictions and TPs (Table 2).

**Table 2.** Biclique prediction performance with different minimum number of compounds (*c*) and proteins (*p*)

Biclique size	# PI	# TP	TPR	# FP	FPR	PPV	TP/PI
*c*: 8, *p*: 4	91	65	0.10	2	0.00	0.97	0.71
*c*: 8, *p*: 3	122	80	0.13	3	0.00	0.96	0.66
*c*: 5, *p*: 3	1295	206	0.33	121	0.01	0.63	0.16
*c*: 5, *p*: 2	1994	248	0.39	171	0.02	0.59	0.12

*Note*: The number of edges represents the number of predicted interactions. PI, predicted interactions, TP/PI provides an estimation of the expected validation success when tested experimentally. Chosen data correspond to bicliques with associated mean FPRs below 0.05, *n*_repeats_ =10. PPV, positive predictive value. Two large bicliques (*c*: 8, *p*: 4/3) and two middle sized bicliques (*c*: 5, *p*: 3/2) are listed, including the biclique size, which was identified as the optimum (*c*: 5, *p*: 2). For a complete data table, see Supplementary Table S3.

#### 3.2.5 Iterative biclique extension verifies specificity

As our method only ever adds positive interactions, but never classifies candidate interactions as negative, it needs to be checked whether iteratively performed cycles would flood the CPI with interactions, leading, in the worst case, to a fully connected biclique network. To test for this scenario, we applied the algorithm in an iterative manner to the STITCH *E.coli* input network with c/p-biclique size set to *c* = 7 and *p* = 3. After 14 iterations, the number of newly predicted interactions fell to below 10, thus essentially, convergence was achieved. During the 14 iterations, the network density increased from 0.02 to 0.04. While the density doubled, it is far from being a fully connected network (density =1). Hence, the biclique-extension approach, even if applied iteratively and performed to convergence, does not lead to an unspecific, fully connected network. Evidently, and as shown above, the choice of *c*/*p*-biclique size critically influences the number of predictions and should be set in dependence of the density of the input network (see Section 4).

### 3.3 Biclique extension performs equally well as complex deep-learning approaches

**Fig. 9.**
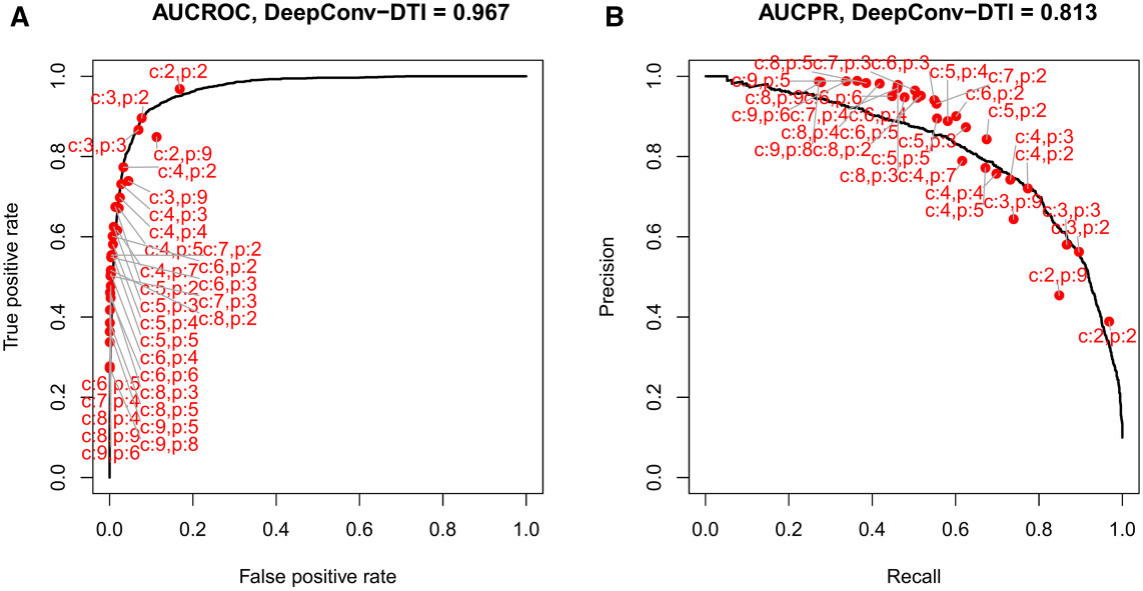
Performance comparison of BiPredict (red circles) and DeepConv-DTI (black solid line) on human test data. (A) TPR-FPR ROC and (B) precision-recall curve. Labels indicate *c*/*p*-biclique size. Note that for BiPredict, no continuous, sortable score variable is produced. Thus, neither a curve nor an associated area can be computed. ROC-curve created using the R-package ROCR (Sing *et al.*, 2005). Areas computed numerically (trapezoid approximation). Label placing by R-package basicPlotteR (Crispell, 2021)

We found the biclique-extension method to perform equally well as, or, regarding some characteristics even better than, advanced, state-of-the-art deep-learning-based methods. We compared BiPredict to the recently published deep-learning method DeepConv-DTI (Lee *et al.*, 2019). DeepConv-DTI uses as input protein sequence information (for proteins) and binary fingerprint vectors that characterize compounds with the regard to a broad range of physico-stereo-chemical properties, and trains a neural network based on positive and negative CPIs. We used the same human input network obtained from STITCH and the same validation data (Liu *et al.*, 2015) for both methods and compared performance with respect to TPR, FPR, and F1-score. The input dataset for DeepConv-DTI was complemented by negative interactions, while BiPredict only needs positive interactions as input. We obtained a TPR of 0.96 with an associated FPR of 0.16 applying biclique extension, allowing a minimum *c*/*p*-biclique size of *c* = 2 and *p* = 2. Using DeepConv-DTI, we obtained a TPR of 0.96 with an associated FPR of 0.23. Biclique extension also performed better than DeepConv-DTI with respect to the F1-score (0.55 versus 0.46). A performance comparison of both methods across the whole range of biclique sizes (BiPredict) and score-values (DeepConv-DTI) reveals a very similar performance of both methods (Fig. 9A) with regard to TPR and FPR (ROC). When inspecting precision versus recall, similar performance characteristics were observed for small *c*/*p*-biclique sizes, but BiPredict performed better taking larger minimum *c*/*p*-biclique sizes (Fig. 9B). Notably, bicliques with many proteins but few compounds as minimum sizes underperform (e.g. *c* = 2, *p* = 9; *c* = 3, *p* = 9).

In addition, we tested for agreement of predictions made by BiPredict, including those for which the answer is not known yet. We tested five non-intersecting subsets of 100 000 predicted interactions, chosen randomly as a test dataset for DeepConv-DTI, after training the model. Of those, DeepConv-DTI predicted an average of 77 469 interactions as positives, which corresponds to an intersection of 77.6% (Supplementary Table S17). Thus, both methods showed high agreement with regard to positive predictions.

### 3.4 KEGG enrichment analysis on the *E.coli* network

Following the performance evaluation, suggesting a high predictive power, we applied the biclique-extension method to the whole *E.coli* STITCH network as input. Applying a confidence input score of 0.4 and setting the biclique-size threshold to the one with detected highest F1-score (*c* = 5, *p* = 2), our method predicts 2666 novel interactions between 127 compounds and 444 proteins. Of note, 681 (25.5%) of those interactions were in fact already contained in the STITCH network, but with an experimental confidence level below our threshold (the complete list of predictions is available as a Supplementary Material).

To inspect the prediction results with regard to biological function (proteins) and their biochemical processes (compounds), we performed a KEGG annotation enrichment analysis on the *E.coli* network to discern metabolic pathways that are enriched in the input STITCH network, in bicliques, and in the predicted interactions. The input list of proteins was compared to the KEGG database as a reference to determine pathway enrichment.

**Fig. 10.**
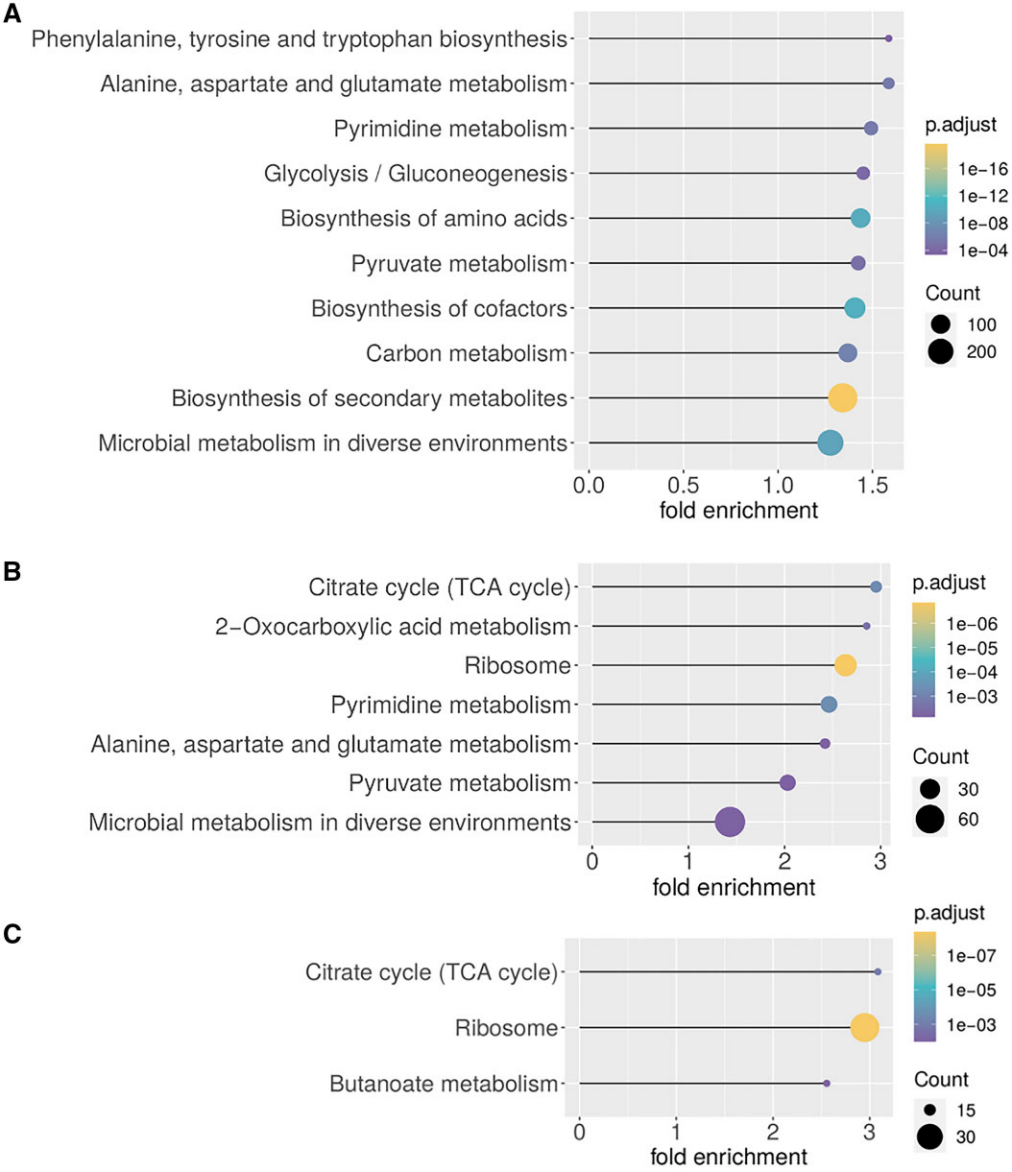
KEGG annotation enrichment analysis of proteins in the *E.coli* network using the R-package clusterProfiler. Shown are up to 10 KEGG-categories with p.adjust < 0.01, ordered by fold-enrichment. Control for multiple testing via the Benjamini–Hochberg method (default in clusterProfiler). (A) Enriched KEGG pathways for all proteins of the input network, (B) for proteins, which are in bicliques up to *c*/*p*-biclique size of *c* = 5 and *p* = 2 (size with highest F1-score) and (C) for proteins of predicted interactions applying the same maximum *c*/*p*-biclique size. For a complete table, see Supplementary Tables S11–S13

In the complete filtered STITCH network of known physical interactions, amino acid metabolism pathways showed the highest fold-enrichment (‘Phenylalanine, tyrosine and tryptophan biosynthesis’ and ‘Alanine, aspartate and glutamate metabolism’) (Fig. 10A) relative to the KEGG annotation. Furthermore, other central metabolic processes (e.g. ‘Glycolysis’), but also the ‘biosynthesis of secondary metabolites’ were found overrepresented, with the latter being associated with the highest significance (smallest adjusted *P*-value). We obtained almost identical results for the set of proteins that were members of at least one biclique (not shown), as almost all proteins were detected to be part of a biclique (all but 10).

The evaluation of our predictions has revealed an optimal maximum *c*/*p*-biclique size of *c* = 5 and *p* = 2. Thus, we additionally analyzed the enrichment in this subset of proteins that belong to bicliques of this optimal maximal size. Here, ‘TCA Cycle’ showed the largest fold-enrichment (Fig. 10B), followed by ‘2-Oxocarboxylic acid metabolism’ and ‘ribosome’. All three were not reported for the full interaction set (Fig. 10A and B). The next four categories reported enriched in bicliques (‘Pyrimidine-’, ‘Alanine, aspirated and glutamate-’, ‘Pyruvate-’ metabolism and ‘Microbial metabolism in diverse environments’) were already reported overrepresented in the whole input network. Thus, with regard to biochemical processes, bicliques show both characteristic as well as common process association.

Next, we analyzed the enrichment for the predicted interactions. These predictions were also calculated based on the bicliques with the maximum *c*/*p*-biqlique size *c* = 5 and *p* = 2. As we predicted new interactions, which are connected to the existing bicliques of the input network, we obtained a similar enrichment as for the input bicliques (overlap of two out of three), with one more process ‘Butanoate metabolism’ reported significant. The largest enriched categories were ‘TCA cycle’ and ‘Ribosome’ (Fig. 10C). A total of 75 out of 420 proteins were only included in the predicted interactions but not part of existing bicliques of the applied *c*/*p*-biclique size.

## 4 Discussion

Aiming to contribute to a deeper understanding of the function of compounds, proteins, and their interactions in metabolic and cellular networks, we searched for the missing links in compound–protein networks with an explicit focus on metabolite–protein interactions. We applied the method of biclique extension, which works by discovering incomplete bicliques in a given network and postulating all edges missing for completion of these bicliques as potentially novel connections. Based only on network topology, we predicted novel interactions in *E.coli* and human CPI networks. Biclique extension needs only one type of information as input and, thus, is easily applicable to all species, for which CPI information is available in reference databases. Biclique extension does not need any detailed knowledge or prediction of binding modes, energetics, and biochemical process involvements. Hence, we believe the biclique-extension method to represent an alternative approach to molecular docking approaches that require molecular structural information and a precise description in interaction potentials and other machine-learning methods (Chen *et al.*, 2016; Tsubaki *et al.*, 2019). As we showed, despite bipartite extension not imposing any molecular information, it does implicitly capture molecular similarity as a determining factor for the validity of inference of interaction (Fig. 5). We believe the primary application scenario of the biclique-extension method to lie in the completion of networks. It can thus be seen as a network curation step. With this study, we have demonstrated that biclique-based methods hold great potential when focusing on metabolite–protein interactions in addition to their successful applications to DTI prediction and drug-repositioning (Lotfi Shahreza *et al.*, 2018).

We achieved a sensitivity of 39% for predictions of CPIs on the STITCH *E.coli* network, while keeping the number of false positives below 5%, which resulted in an associated precision of 59% (Fig. 6A and Supplementary Table S3). We demonstrated an even better prediction performance for the human STITCH network, in which we obtained a maximum sensitivity of nearly 78%, increasing with the total number of predicted interactions (Fig. 6C and Supplementary Table S7).

Even higher prediction performance was shown for both organisms using the KEGG metabolic-reaction network as input. Here, a sensitivity of 40% with an associated precision of 81% was obtained for *E.coli* (Fig. 8A and Supplementary Table S18) and a sensitivity of 84% with an associated precision of 88% for human (Fig. 8C and Supplementary Table S20). The performance for human based on the BDB network was similar to the performance based on the STITCH network, with a sensitivity of 77% and an associated precision of 72% (Supplementary Fig. S6 and Table S16).

In both species and all tested networks, prediction performance levels were significantly and substantially above random predictions (Figs 6 and 8). We found that performance rates depended on the chosen *c*/*p*-biclique sizes. The best sensitivity for all networks was obtained when including smaller *c*/*p*-biclique sizes, even though larger bicliques occur at higher frequency in the human STITCH network than in the *E.coli* STITCH network. The larger the chosen *c*/*p*-biclique sizes, the larger the set of compounds and proteins with known interactions captured by these bicliques. As expected, this resulted in an increase of precision as well as a decrease of FPRs, as the logic of the biclique extension rests on more support (Figs 6 and 8). In this regard, we also attribute the better performance in human to the presence of larger bicliques in the input network (Fig. 4B). However, large *c*/*p*-biclique sizes also lead to a smaller number of bicliques in the input network, and, thus, result in a lower total number of predicted interactions and lower sensitivity/recall (Figs 6, 8 and 9 and Supplementary Tables S3, S4, S7 and S8). For the *E.coli* and the human STITCH network, we found the optimal minimum *c*/*p*-biclique sizes were of four or five compounds and two proteins, with regard to maximum TPRs and minimum FPRs. Nevertheless, different *c*/*p*-biclique sizes should be tested on used input networks. Another effective filter criterion would be to limit the total number of predicted interactions, as this also keeps the FPRs low. Consistently, we found best performance for bicliques with more compounds than proteins in them (Figs 6, 8 and 9). As this holds also for the frequencies of biclique sizes for the input network (Fig. 4), we assume this to be purely a statistical effect. Comparing the STITCH and the KEGG networks, the prediction performances and the network properties regarding their density, we can conclude, that for input networks with relatively high density, like the STITCH *E.coli* network, the minimum *c*/*p*-biclique size should be chosen at higher values (at least *c* = 4/5, *p* = 2), while for lower density input networks (like all other networks studied here) also a smaller *c*/*p*-biclique size (even *c* = 2, *p* = 2) seems optimal.

By its very nature, our method predicts missing links in CPI networks, but never classifies candidate interactions as negative. To test whether this would eventually lead to a fully connected network, we iteratively applied the biclique algorithm to the network, which converged at a doubled network density (from 0.02 to 0.04), which was far from a saturated network (density =1). Thus, we could show that BiPredict does not indiscriminately ‘flood’ a network with interactions, but is rather specific. Also biologically, positive interactions are of greater interest than non-interactions. The binary yes/no classification of interactions is a simplification anyway, as actually, gradual differences of binding energies determine the strengths of interactions and concentrations the probabilities thereof.

Evidently, bipartite extension depends on the input network to be correct. While this can be assumed to be true for the used networks—within the limits of the employed confidence assessment—we also found that the biclique extension proved robust with regard to the chosen STITCH confidence scores (Fig. 7). This may reveal a strength of the biclique-extension method. It integrates over many interactions to make predictions and may thus prove error tolerant. In addition, concordant results were obtained using different input databases (BDB, see Section 3.2.2, and KEGG, see Section 3.2.3).

We compared the obtained performance rates for the *E.coli* network to rates from a recent experimental study (*E.coli* CPIs) (Piazza *et al.*, 2018) to evaluate whether our approach would be able to improve the prediction of novel CPIs in general. Indeed, focusing tests on predicted interactions obtained by the biclique-extension method would increase the fraction of validated interactions in relation to the number of tests. In detail, Piazza *et al.* tested 34 186 interactions experimentally and reported 1719 validated interactions, out of which 1487 were novel targets. This reflects a validation rate of 0.04 for novel interactions and 0.05 relative to all validated interactions (including also previously known interactions). Our predictions resulted in mean validation rates of 12% with TPRs of 39% up to validation rates of 71% with TPRs of 10%, relative to the number of predicted interactions and with FPRs below 5% (*n* = 10). Noticeably, we can even assume these rates to actually be higher, as several of the predicted interactions might contain additional TP interactions that are not yet part of the set of real positive cases found by experimental testing. Clearly, Piazza *et al.* went for systematic testing and did not aim to optimize for the highest rate of validated interactions. However, achieving an increase of validated interactions by simultaneously reducing the number of required tests would save time and resources. Consequently, novel interaction partners predicted by biclique extension can be highly supportive when considered for experimental testing.

We also compared BiPredict performance rates to those of the recently published deep learning method DeepConv-DTI (Lee *et al.*, 2019), which itself was compared to several state-of-the-art approaches in the respective study. We found the biclique-extension method to perform equally well as, or, regarding some characteristics, even better than advanced, state-of-the-art deep-learning-based methods (see Section 3.3).

Noticeably, the number of interactions predicted by BiPredict in the human STITCH network was very high (up to 1.5 million, depending on *c*/*p*-biclique size) in relation to the input network size (*n* = 44 322). However, these represented only 10% of the 15.9 million potential interactions given the number of compounds and proteins in the network. To further validate these seemingly high numbers of predicted interactions, we tested five subsets of BiPredict-predicted interactions for agreement with DeepConv-DTI and obtained a high average overlap of 77.6% predicted positive interactions, which were concordantly predicted by DeepConv-DTI and BiPredict. Thus, the predictions made by BiPredict were supported by other methods (DeepConv-DTI) as well. Please note that both methods also predict false positives, such that an overlap of 100% cannot be expected.

When studying metabolite–protein interactions, two different modes of binding need to be contemplated. That of a metabolite binding as a substrate and that of a metabolite binding as an allosteric modulator. For the latter, approaches developed for ‘conventional’ CPIs can readily be transferred. For the former, the multi-molecular nature of substrate binding, combined with biochemical conversions, renders the applicability of single compound–protein binding prediction methods questionable. Here, we showed that for both modes, biclique extension generates meaningful results. When using STITCH and BDB as input networks for predictions, classical compound–protein binding events can be considered captured (Fig. 6 and Supplementary Fig. S6), as both focus on compound-binding, irrespective of metabolic conversions. Using a KEGG-reaction network as input, we also obtained high-confidence predictions (Fig. 8). Thus, we conclude that the rationale of biclique extensions works equally well for both scenarios: single-molecule binding as an effector and binding as a substrate.

Biclique extension can only be applied to those interactions, for which its rationale applies. Isolated interactions of one compound and one protein, e.g. cannot be captured and are, for principal reasons, not predictable by BiPredict, even when applying the smallest considered biclique size (*c* = 2, *p* = 2). However, judging from the STITCH input CPI, this constitutes only a small fraction of ‘dark’ CPIs relative to all interactions (7.8% in *E.coli* and 4.9% in human, Table 1).

With regard to biochemical process involvement of compounds and proteins represented in bicliques and biclique-based interaction predictions, we found TCA-cycle, ribosome overrepresented in both (Fig. 10B and C). As the TCA-cycle is a central biochemical integration hub with relatively tight metabolites–enzyme interactions described before (albeit in yeast) (Durek and Walther, 2008), the appearance of this process in this statistic seems very plausible. Furthermore, amongst TCA-enzymes/proteins themselves, many interactions have been reported (in *Arabidopsis thaliana*) (Zhang *et al.*, 2018), rendering an associated compound–protein biclique interactions more likely, with the notion of support of substrate channeling referred to as metabolons observed for TCA-cycle. Of particular interest here are the reported new interactions within the TCA-cycle (see Supplementary Material) that await experimental verification. By contrast, as the ribosome is not immediately considered associated with metabolism, but with translation, its appearance seems surprising. However, as the ribosome is a multiprotein complex with many proteins binding to co-factors, such as ATP and ADP, a densely knit interaction network with associated predictions can be explained as well. Of note also, even when probing the whole input STITCH interaction network relative to the KEGG annotation, pronounced overrepresentation of a number of metabolic functions have been observed (Fig. 10A). Thus, physical interactions form a much denser network than that of biochemical reaction-based substrate/product–enzyme interactions captured by KEGG.

In summary, we demonstrated that biclique extension is indeed an effective approach for the prediction of CPIs in naturally occurring metabolic and cellular networks. Biclique extension works under minimal assumptions that are solely inferred from observed interaction networks and their topology and is not limited by the currently known biochemical and physico-chemical determinants of CPIs, which offers a great potential to find novel interaction candidates and to support efficient experimental testing. The biclique-extension methodology can be readily applied to all species with available CPI interactions. With this study, we provide a basis that allows choosing the parameters of biclique extension-based predictions and provide expected performance levels of their applications in the context of metabolite–protein interaction networks.

## Authors’ contributions

D.W. devised and supervised the study. S.T. collected and processed the data, planned and implemented analyses and performed all computations, including software development and statistical analyses. S.T. and D.W. analyzed the data and interpreted the results, and wrote the manuscript.

## Funding

This work was supported by the Max Planck Society.

*Conflict of Interest*: none declared.

## Supplementary Material

vbac001_Supplementary_Data

## References

[vbac001-B1] Bleakley K. , Yamanishi Y. (2009) Supervised prediction of drug–target interactions using bipartite local models. Bioinformatics, 25, 2397–2403. 19605421 10.1093/bioinformatics/btp433 PMC2735674

[vbac001-B2] Bleakley K. et al (2007) Supervised reconstruction of biological networks with local models. Bioinformatics, 23, i57–i65. 17646345 10.1093/bioinformatics/btm204

[vbac001-B3] Cannistraci C.V. et al (2013) From link-prediction in brain connectomes and protein interactomes to the local-community-paradigm in complex networks. Sci. Rep., 3, 1613. 23563395 10.1038/srep01613 PMC3619147

[vbac001-B4] Chen L. et al (2016) Identification of compound–protein interactions through the analysis of gene ontology, KEGG enrichment for proteins and molecular fragments of compounds. Mol. Genet. Genomics, 291, 2065–2079. 27530612 10.1007/s00438-016-1240-x

[vbac001-B5] Crispell J. (2021) basicPlotteR: a collection of functions to help with base R plotting. R package version 0.0.0.9000.

[vbac001-B6] Csardi G. , Nepusz T. (2006) The igraph software package for complex network research. Int. J. Complex Syst., 1695, 1–9.

[vbac001-B7] Daminelli S. et al (2012) Drug repositioning through incomplete bi-cliques in an integrated drug-target-disease network. Integr. Biol. (Camb), 4, 778–788. 22538435 10.1039/c2ib00154c

[vbac001-B8] Daminelli S. et al (2015) Common neighbours and the local-community-paradigm for topological link prediction in bipartite networks. New J. Phys., 17, 113037.

[vbac001-B9] Diether M. et al (2019) Systematic mapping of protein-metabolite interactions in central metabolism of *Escherichia coli*. Mol. Syst. Biol., 15, e9008. 31464375 10.15252/msb.20199008 PMC6706640

[vbac001-B10] Durek P. , Walther D. (2008) The integrated analysis of metabolic and protein interaction networks reveals novel molecular organizing principles. BMC Syst. Biol., 2, 100. 19032748 10.1186/1752-0509-2-100 PMC2607255

[vbac001-B11] Eslami Manoochehri H. , Nourani M. (2020) Drug-target interaction prediction using semi-bipartite graph model and deep learning. BMC Bioinformatics, 21, 248. 32631230 10.1186/s12859-020-3518-6 PMC7336396

[vbac001-B12] Fujibuchi W. et al (1998) DBGET/LinkDB: an integrated database retrieval system. Pac. Symp. Biocomput., 98, 683–694. 9697222

[vbac001-B13] Gilson M.K. et al (2016) BindingDB in 2015: a public database for medicinal chemistry, computational chemistry and systems pharmacology. Nucleic Acids Res., 44, D1045–D1053. 26481362 10.1093/nar/gkv1072 PMC4702793

[vbac001-B14] Giri V. et al (2015) RxnSim: a tool to compare biochemical reactions. Bioinformatics, 31, 3712–3714. 26187943 10.1093/bioinformatics/btv416

[vbac001-B15] Gobbi A. et al (2020) BiRewire: high-performing routines for the randomization of a bipartite graph (or a binary event matrix), undirected and directed signed graph preserving degree distribution (or marginal totals). Bioconductor version: Release (3.10).

[vbac001-B16] Guha R. (2007) Chemical informatics functionality in R. J. Stat. Softw., 18, 1–16.

[vbac001-B17] Kanehisa M. et al (2017) KEGG: new perspectives on genomes, pathways, diseases and drugs. Nucleic Acids Res., 45, D353–D361. 27899662 10.1093/nar/gkw1092 PMC5210567

[vbac001-B18] Kuhn M. et al (2008) STITCH: interaction networks of chemicals and proteins. Nucleic Acids Res., 36, D684–D688. 18084021 10.1093/nar/gkm795 PMC2238848

[vbac001-B19] Lee I. et al (2019) DeepConv-DTI: prediction of drug-target interactions via deep learning with convolution on protein sequences. PLoS Comput. Biol., 15, e1007129. 31199797 10.1371/journal.pcbi.1007129 PMC6594651

[vbac001-B20] Lim S. et al (2021) A review on compound-protein interaction prediction methods: data, format, representation and model. Comput. Struct. Biotechnol. J., 19, 1541–1556. 33841755 10.1016/j.csbj.2021.03.004 PMC8008185

[vbac001-B21] Lima-Mendez G. , Helden J.v. (2009) The powerful law of the power law and other myths in network biology. Mol. Biosyst., 5, 1482–1493. 20023717 10.1039/b908681a

[vbac001-B22] Liu H. et al (2015) Improving compound–protein interaction prediction by building up highly credible negative samples. Bioinformatics, 31, i221–i229. 26072486 10.1093/bioinformatics/btv256 PMC4765858

[vbac001-B23] Lotfi Shahreza M. et al (2018) A review of network-based approaches to drug repositioning. Brief Bioinform., 19, 878–892. 28334136 10.1093/bib/bbx017

[vbac001-B24] Lu Y. et al (2020) Biclique: an R package for maximal biclique enumeration in bipartite graphs. BMC Res. Notes, 13, 88. 32085812 10.1186/s13104-020-04955-0 PMC7035696

[vbac001-B25] Piazza I. et al (2018) A map of protein-metabolite interactions reveals principles of chemical communication. Cell, 172, 358–372.e23. 29307493 10.1016/j.cell.2017.12.006

[vbac001-B26] R Core Team. (2016) R: a language and environment for statistical computing. Vienna, Austria.

[vbac001-B27] Schweiger R. et al (2011) Generative probabilistic models for protein–protein interaction networks—the biclique perspective. Bioinformatics, 27, i142–i148. 21685063 10.1093/bioinformatics/btr201 PMC3117378

[vbac001-B28] Sing T. et al (2005) ROCR: visualizing classifier performance in R. Bioinformatics, 21, 3940–3941. 16096348 10.1093/bioinformatics/bti623

[vbac001-B29] Szklarczyk D. et al (2016) STITCH 5: augmenting protein–chemical interaction networks with tissue and affinity data. Nucleic Acids Res., 44, D380–D384. 26590256 10.1093/nar/gkv1277 PMC4702904

[vbac001-B30] Szklarczyk D. et al (2019) STRING v11: protein-protein association networks with increased coverage, supporting functional discovery in genome-wide experimental datasets. Nucleic Acids Res., 47, D607–D613. 30476243 10.1093/nar/gky1131 PMC6323986

[vbac001-B31] The UniProt Consortium (2017) UniProt: the universal protein knowledgebase. Nucleic Acids Res., 45, D158–D169. 27899622 10.1093/nar/gkw1099 PMC5210571

[vbac001-B32] Torchiano M. (2016) Effsize - a package for efficient effect size computation Zenodo.

[vbac001-B33] Tsubaki M. et al (2019) Compound–protein interaction prediction with end-to-end learning of neural networks for graphs and sequences. Bioinformatics, 35, 309–318. 29982330 10.1093/bioinformatics/bty535

[vbac001-B34] Wu T. et al (2021) clusterProfiler 4.0: a universal enrichment tool for interpreting omics data. Innovation, 2, 100141. 34557778 10.1016/j.xinn.2021.100141 PMC8454663

[vbac001-B35] Wu Z. et al (2018) Network-based methods for prediction of drug-target interactions. Front. Pharmacol., 9, 1134. 30356768 10.3389/fphar.2018.01134 PMC6189482

[vbac001-B36] Yu G. et al (2012) clusterProfiler: an R package for comparing biological themes among gene clusters. OMICS, 16, 284–287. 22455463 10.1089/omi.2011.0118 PMC3339379

[vbac001-B37] Zhang Y. et al (2014) On finding bicliques in bipartite graphs: a novel algorithm and its application to the integration of diverse biological data types. BMC Bioinformatics, 15, 110. 24731198 10.1186/1471-2105-15-110 PMC4038116

[vbac001-B38] Zhang Y. et al (2018) The extra-pathway interactome of the TCA cycle: expected and unexpected metabolic interactions. Plant Physiol., 177, 966–979. 29794018 10.1104/pp.17.01687 PMC6052981

