# Supplementary material for: Biclique extension as an effective approach to identify missing links in metabolic compound–protein interaction networks
Source: Bioinform Adv. 2022 Jan 12;2(1):vbac001. doi: 10.1093/bioadv/vbac001 (PMC9710583; doi:10.1093/bioadv/vbac001)
Supplement: vbac001_Supplementary_Data [file vbac001_supplementary_data.zip › Suppl of Biclique Extension Manuscript.pdf]

Biclique extension as an effective approach to identify missing links in metabolic compound-protein interaction networks.

Sandra Thieme and Dirk Walther

Supplementary information

Supplementary Figures

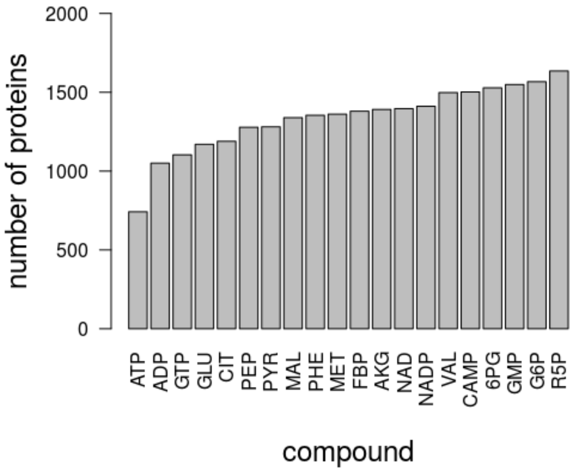

Supplementary Figure 1*. Reported number of negative interactions (no interaction observed) for the 20 compounds contained in the Piazza.negatives (Supplementary table 1).*

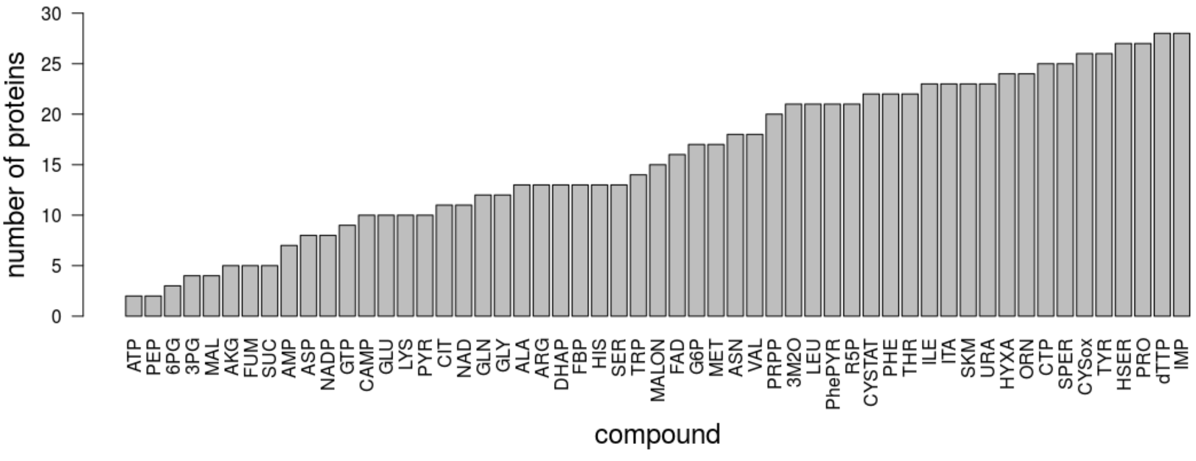

Supplementary Figure 2*. Reported number of negative interactions (no interaction observed) for the 55 compounds contained in the Diether.negatives (Supplementary table 2).*

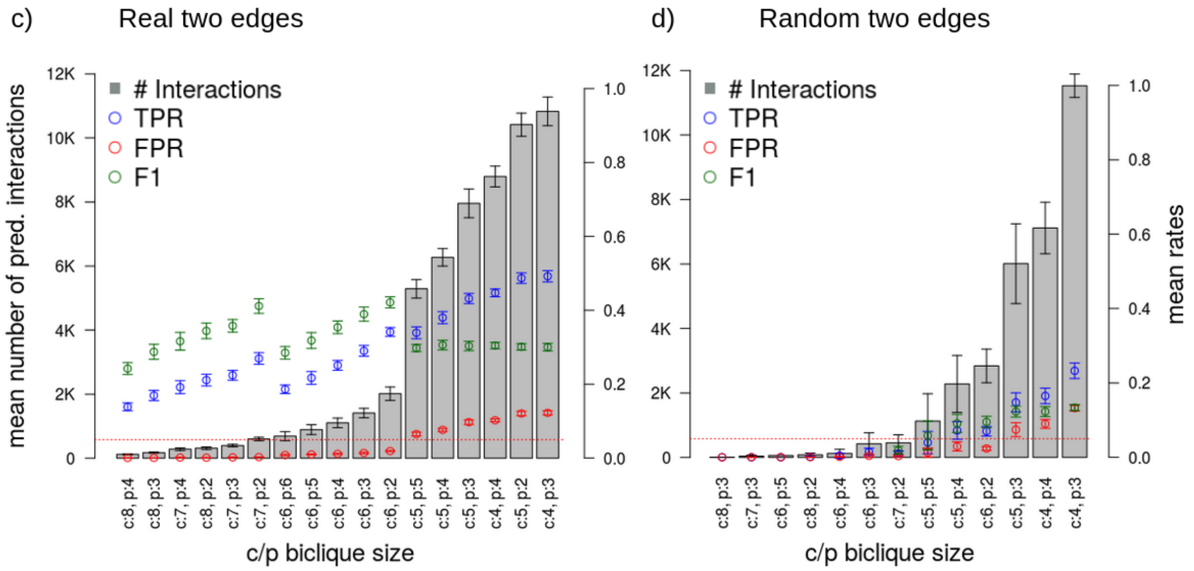

Supplementary Figure 3. *Results of the biclique-extension-based interaction predictions using a confidence threshold of 0.4 and with ten repetitions on different random samples (10% removed true interactions). With insertion of one edge (a,b) and insertion of two edes (c,d) for larger bicliques (minimum of four nodes on the side to which interactions are added). On the left-hand side, the results for the real network are shown (a,c) and on the right-hand side the results for the randomized network (b,d). Sorting of data corresponding to different biclique sizes in ascending order of the number of predicted interactions (grey bars, average of 10 runs). The best obtained biclique size under the one-edge addition scenaria was c = 5 and p = 2, with maximal TPR with concurrent FPR<0.05 and highest F1-score. For the two-edge addition mode, c=6, p=2 proved optimal. The red dotted line marks the 0.05-line to allow for better visual clarity with regard to FPR. Note that the shown c/p biclique sizes represent the subset of all possible biclique sizes resulting in less than 12,000 predicted interactions, i.e. about twice the number of interactions in the input reference network. (Supplementary Tables 5, 6). Left ordinate axis: number of predicted interactions, right ordinate: rates representing fractional values of TPR, FPR, and F1. Error bars correspond to standard deviations.*

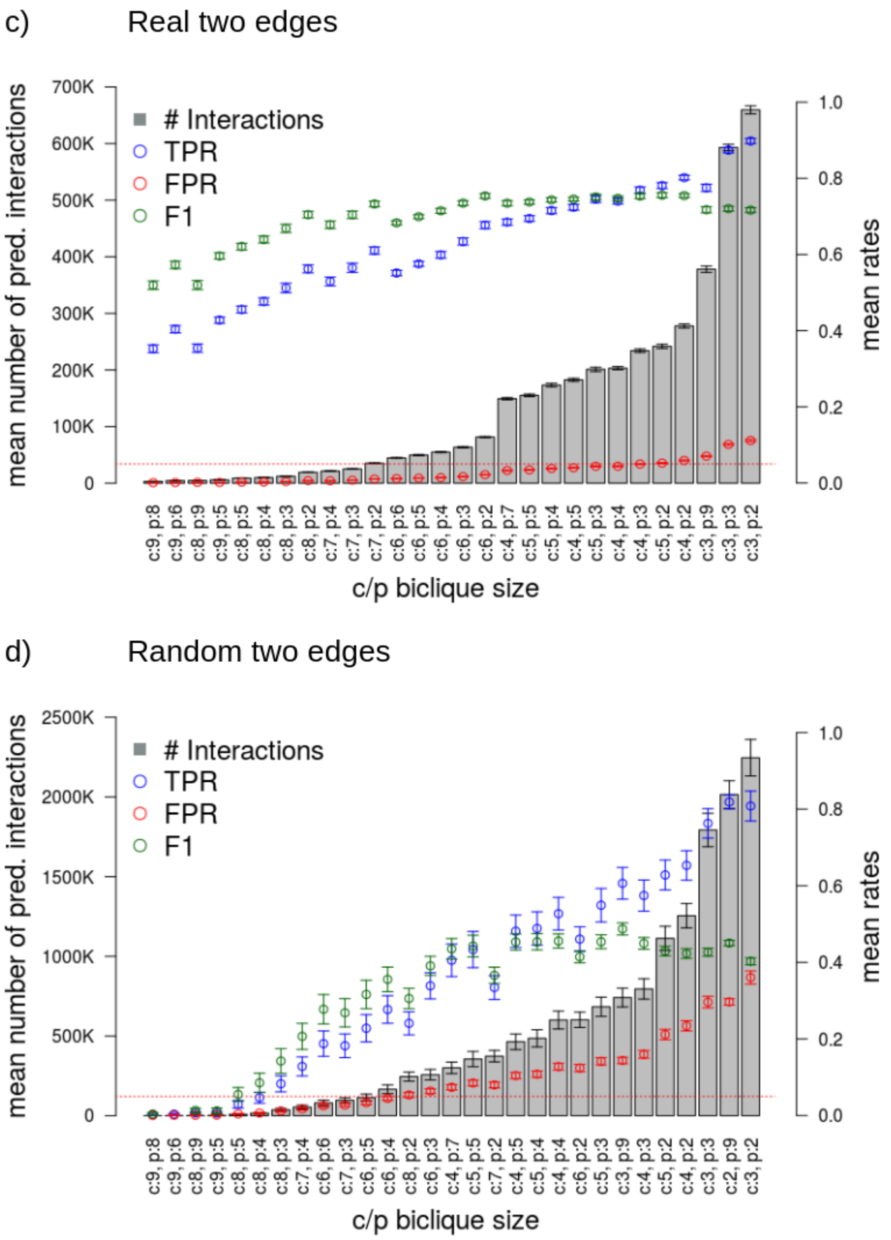

Supplementary Figure 4. *Results of the biclique-extension-based interaction predictions using a confidence threshold of 0.4 and with ten repetitions on different random samples (5% removed true interactions). With insertion of two edes for larger bicliques (minimum of four nodes on the side to which interactions are added). Sorting of data corresponding to different biclique sizes in ascending order of the number of predicted interactions (grey bars, average of 10 runs). The best obtained biclique size under the two-edge addition mode was c=6, p=2, with highest F1. The red dotted line marks the 0.05-line to allow for better visual clarity with regard to FPR. Note that the shown c/p biclique sizes represent the subset of all possible biclique sizes resulting in less than 700,000 predicted interactions for real data and comparable biclique size ranges for random data. (Supplementary Table 9-10). Left ordinate axis: number of predicted interactions, right ordinate: rates representing fractional values of TPR, FPR, and F1. Error bars correspond to standard deviations*

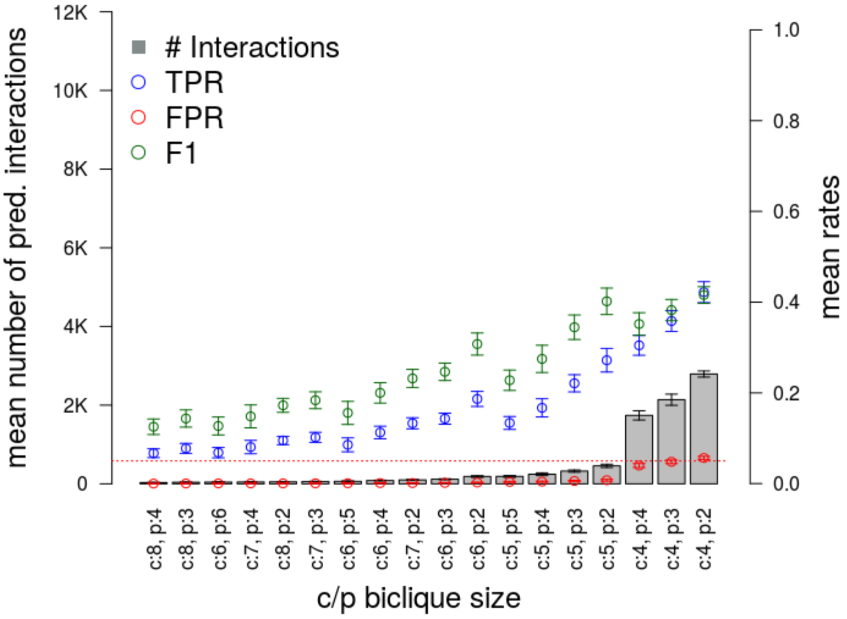

Supplementary Figure 5. *Results of biclique-extension-based interaction prediction for E. coli after removing currency metabolites (see Supplementary table 9) from the network and using a confidence threshold of 0.4 and with ten repetitions on different random samples. Insertion of one edge was allowed for predictions. Sorting of data corresponding to different biclique sizes in ascending order of the number of predicted interactions (grey bars, average of 10 runs). The best obtained biclique size under the one-edge addition scenaria was c = 4/5 and p = 2, with maximal TPR with concurrent FPR<0.05. Note that the shown c/p biclique sizes represent the subset of all possible biclique sizes resulting in less than 12,000 predicted interactions, i.e. about twice the number of interactions in the input reference network. Left ordinate axis: number of predicted interactions, right ordinate: rates representing fractional values of TPR, FPR, and F1.*

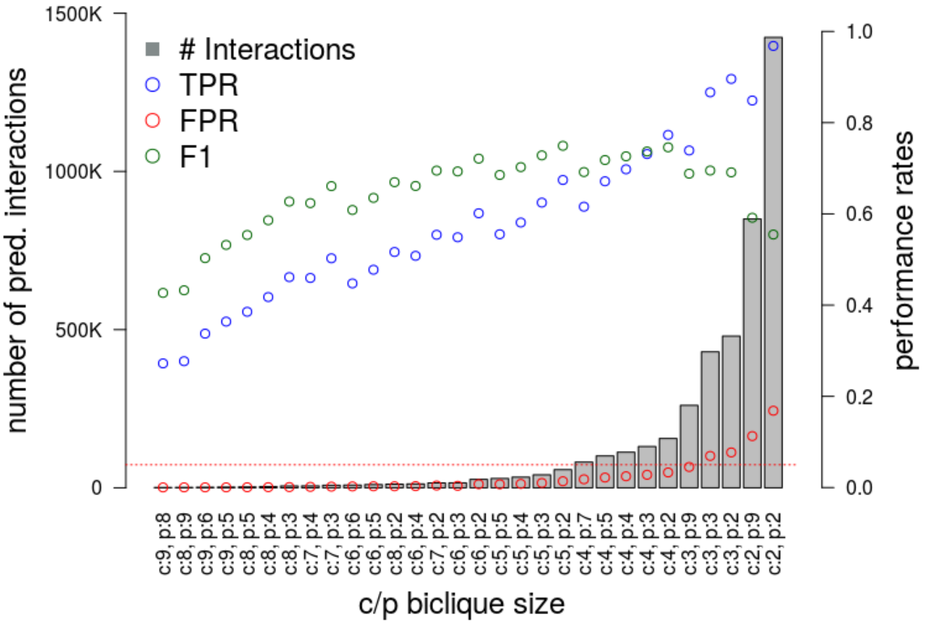

Supplementary Figure 6. *Results of the biclique-extension-based interaction predictions using the BindingDB input network (5% removed true interactions). Sorting of data corresponding to different biclique sizes in ascending order of the number of predicted interactions. The best obtained biclique sizes were c = 5/4 and p = 2, with maximal TPR with concurrent TPR<0.05 and highest F1-score. The red dotted line marks the 0.05-line to allow for better visual clarity with regard to FPR. (Supplementary Table 16). Left ordinate axis: number of predicted interactions, right ordinate: rates representing fractional values of TPR, FPR, and F1.*

Supplementary Tables

Supplementary Table 1. *List of all 20 compounds included in the validation dataset of Piazza et al (Piazza et al. 2018).*

**Abbreviation**	**Name**	**KEGG ID**
ATP	Adenosine triphosphate	C00002
NAD	Nicotinamide adenine dinucleotide	C00003
NADP	Nicotinamide adenine dinucleotide phosphate	C00006
ADP	Adenosine diphosphate	C00008
Pyr	Pyruvate	C00022
L-Gln	L-Glutamate	C00025
AKG	2-Oxoglutarate	C00026
GTP	Guanosine triphosphate	C00044
L-Met	L-Methionine	C00073
PEP	Phosphoenolpyruvate	C00074
L-Phe	L-Phenylalanine	C00079
G6P	D-Glucose 6-phosphate	C00092
R5P	alpha-D-Ribose 5-phosphate	C00117
GMP	Guanosine monophosphate	C00144
Mal	L-Malate	C00149
Cit	Citrate	C00158
L-Val	L-Valine	C00183
PGP	6-Phospho-D-gluconate	C00345
FBP	D-Fructose 1,6-bisphosphate	C00354
cAMP	3',5'-cyclic adenosine monophosphate	C00575

Supplementary Table 2*. List of all compounds included in the validation dataset of Diether et al (Diether et al. 2019).*

Abbreviation	Name	KEGG ID
3M2O	2-Ketoisovalerate, 2-Oxoisovalerate	C00141
AKG	Ketoglutarate	C00026
THR	Threonine	C00188
G6P	alpha-D-glucose 6-phosphate	C00092
AMP	Adenosine monophosphate	C00020
ARG	Arginine	C00062
ASN	Asparagine	C00152
ATP	MgATP	C00002
CAMP	cAMP	C00575
CIT	Citrate	C00158
ORN	L-Ornithine	C00077
6PG	6-Phosphogluconic acid	C00345
ASP	Aspartate	C00049
MAL	L-Malate	C00149
CTP	ara-CTP	C00063
CYSox	Cysteine	C00097
ALA	Alanine	C00041
GLU	Glutamate	C00025
PRO	Proline	C00148
SER	D-Serine	C00065
DHAP	Dihydroxyacetone phosphate	C00111
FAD	Flavin adenine dinucleotide	C00016
FBP	Fructose-1,6-diphosphate	C00354
FUM	Fumarate	C00122
3PG	3-Phosphoglycerate	C00197
GLN	Glutamine	C00064
GLY	Glycine	C00037
GTP	Guanosine triphosphate	C00044
HIS	Histidine	C00135

HSER	L-Homoserine	C00263
HYXA	Hypoxanthine	C00262
ILE	Isoleucine	C00407
IMP	Inosine monophosphate	C00130
ITA	Itaconate	C00490
CYSTAT	L-Cystathionine	C02291
LEU	Leucine	C00123
LYS	Lysine	C00047
MALON	Malonate	C00383
MET	Methionine	C00073
NAD	Diphosphopyridine nucleotide	C00003
NADP	Nicotinamide adenine dinucleotide phosphate	C00006
PHE	Phenylalanine	C00079
PhePYR	Phenylpyruvate	C00166
PEP	Phosphoenolpyruvate	C00074
PRPP	5-Phosphoribosyl-1-pyrophosphate	C00119
PYR	Pyruvate	C00022
SKM	Shikimic acid	C00493
SPER	Spermidine	C00315
SUC	Succinate	C00042
TRP	Tryptophan	C00078
dTTP	Thymidine 5'-	C00459
TYR	Tyrosine	C00082
URA	Uracil	C00106
VAL	L-Valine	C00183
R5P	Riboside 5-monophosphate	C00117

Supplementary Table 3. *In relation to Figure 6a. Statistical results of biclique calculations for E. coli with different minimum number of compounds (c) and proteins (p) on each side of the biclique using a confidence threshold of 0.4 and allowing insertion of one edge to complete a biclique. The number of edges represents the number of predicted interactions. Datasets are sorted according to the number of edges. All values are the mean values of n=10 repetitions. Note that the underlying reference data was unbalanced with a mean number of positives n= 630 and a mean number of negatives n = 9,116. Dataset with maximum F1 score referred to in the text is highlighted.*

Dataset	# Edges	# TP	TPR	# FP	FPR	PPV	F1
Biclique c:8, p:4	91	65	0.10	2	0.00	0.97	0.19
Biclique c:8, p:3	122	80	0.13	3	0.00	0.96	0.22
Biclique c:7, p:4	137	92	0.15	4	0.00	0.97	0.25
Biclique c:6, p:6	157	87	0.14	9	0.00	0.91	0.24
Biclique c:7, p:3	202	118	0.19	6	0.00	0.95	0.31
Biclique c:8, p:2	205	104	0.17	7	0.00	0.94	0.28
Biclique c:6, p:5	214	103	0.16	10	0.00	0.91	0.28
Biclique c:6, p:4	288	128	0.20	14	0.00	0.90	0.33
Biclique c:7, p:2	335	145	0.23	12	0.00	0.93	0.37
Biclique c:6, p:3	404	154	0.25	20	0.00	0.89	0.38
Biclique c:6, p:2	664	188	0.30	34	0.00	0.85	0.44
Biclique c:5, p:5	761	145	0.23	81	0.01	0.64	0.34
Biclique c:5, p:4	977	178	0.28	95	0.01	0.65	0.39
Biclique c:5, p:3	1,295	206	0.33	121	0.01	0.63	0.43
**Biclique c:5, p:2**	**1,994**	**248**	**0.39**	**171**	**0.02**	**0.59**	**0.47**
Biclique c:4, p:4	6,943	270	0.43	746	0.08	0.27	0.33
Biclique c:4, p:3	8,428	299	0.47	887	0.10	0.25	0.33
Biclique c:4, p:2	10,683	338	0.54	1,047	0.11	0.24	0.33
Biclique c:3, p:3	42,962	449	0.71	4,142	0.45	0.10	0.17
Biclique c:3, p:2	48,039	470	0.75	4,342	0.48	0.10	0.17
Biclique c:2, p:2	136,643	604	0.96	8,119	0.89	0.07	0.13

Supplementary Table 4. *In relation to Figure 6b. Statistical results of biclique calculations for E. coli randomized network with different minimum number of compounds (c) and proteins (p) on each side of the biclique using a confidence threshold of 0.4 and allowing insertion of one edge. The number of edges represents the number of predicted interactions. Datasets are sorted according to the number of edges. All values are the mean values of n=10 repetitions. Note that the underlying reference data was unbalanced with a mean number of positives n= 503 and a mean number of negatives n = 8,586.*

Dataset	# Edges	# TP	TPR	# FP	FPR	PPV	F1
Biclique c:7, p:3	2	0	0.00	0	0.00	NaN	NaN
Biclique c:6, p:4	5	0	0.00	1	0.00	NaN	NaN
Biclique c:6, p:3	50	1	0.00	5	0.00	NaN	NaN
Biclique c:8, p:2	83	1	0.00	4	0.00	0.13	NaN
Biclique c:5, p:5	156	4	0.01	18	0.00	NaN	NaN
Biclique c:5, p:4	417	9	0.02	44	0.01	0.18	0.03
Biclique c:7, p:2	438	5	0.01	23	0.00	0.16	0.02
Biclique c:5, p:3	1,120	17	0.03	111	0.01	0.14	0.05
Biclique c:6, p:2	1,698	18	0.04	85	0.01	0.18	0.06
Biclique c:5, p:2	5,796	53	0.11	382	0.04	0.12	0.11
Biclique c:4, p:4	7,402	79	0.16	813	0.09	0.09	0.11
Biclique c:4, p:3	11,875	108	0.22	1,174	0.14	0.09	0.12
Biclique c:4, p:2	21,210	153	0.30	1,657	0.19	0.08	0.13
Biclique c:3, p:3	61,568	303	0.60	4,953	0.58	0.06	0.11
Biclique c:3, p:2	74,844	321	0.64	5,278	0.61	0.06	0.11
Biclique c:2, p:2	158,768	466	0.93	8,035	0.94	0.05	0.10

Supplementary Table 5. *In relation to Supplementary Figure 3c. Statistical results of biclique calculations for E. coli with different minimum number of compounds (c) and proteins (p) on each side of the biclique using a confidence threshold of 0.4 and allowing insertion of two edges. The number of edges represents the number of predicted interactions. Datasets are sorted according to the number of edges. All values are the mean values of n=10 repetitions. Note that the underlying reference data was unbalanced with a mean number of positives n= 624 and a mean number of negatives n = 9,089. Dataset with maximum F1 score referred to in the text is highlighted.*

Dataset	# Edges	# TP	TPR	# FP	FPR	PPV	F1
Biclique c:8, p:4	122	86	0.14	3	0.00	0.96	0.24
Biclique c:8, p:3	175	106	0.17	6	0.00	0.95	0.29
Biclique c:7, p:4	283	120	0.19	13	0.00	0.90	0.32
Biclique c:8, p:2	315	132	0.21	9	0.00	0.94	0.34
Biclique c:7, p:3	398	140	0.22	17	0.00	0.89	0.36
Biclique c:7, p:2	602	168	0.27	25	0.00	0.87	0.41
Biclique c:6, p:6	690	116	0.19	76	0.01	0.61	0.28
Biclique c:6, p:5	894	136	0.22	91	0.01	0.60	0.32
Biclique c:6, p:4	1,104	157	0.25	103	0.01	0.60	0.35
Biclique c:6, p:3	1,412	181	0.29	126	0.01	0.59	0.39
**Biclique c:6, p:2**	**2,015**	**213**	**0.34**	**174**	**0.02**	**0.55**	**0.42**
Biclique c:5, p:5	5,290	212	0.34	590	0.07	0.27	0.30
Biclique c:5, p:4	6,272	237	0.38	694	0.08	0.26	0.31
Biclique c:5, p:3	7,956	270	0.43	883	0.10	0.23	0.30
Biclique c:4, p:4	8,796	279	0.45	930	0.10	0.23	0.30
Biclique c:5, p:2	10,414	304	0.49	1,097	0.12	0.22	0.30
Biclique c:4, p:3	10,828	307	0.49	1,112	0.12	0.22	0.30
Biclique c:4, p:2	13,877	347	0.56	1,326	0.15	0.21	0.30
Biclique c:3, p:3	43,529	447	0.72	4,096	0.45	0.10	0.17
Biclique c:3, p:2	48,783	472	0.76	4,369	0.48	0.10	0.18
Biclique c:2, p:2	137,270	600	0.96	8,099	0.89	0.07	0.13

Supplementary Table 6. *In relation to Supplementary Figure 3d. Statistical results of biclique calculations for E. coli randomized network with different minimum number of compounds (c) and proteins (p) on each side of the biclique using a confidence threshold of 0.4 and allowing insertion of one edge. The number of edges represents the number of predicted interactions. Datasets are sorted according to the number of edges. All values are the mean values of n=10 repetitions. Note that the underlying reference data was unbalanced with mean number of positives n= 508 and the mean number of negatives n = 8,585.*

Dataset	# Edges	# TP	TPR	# FP	FPR	PPV	F1
Biclique c:8, p:3	10	0	0.00	0	0.00	NaN	NaN
Biclique c:7, p:3	40	0	0.00	2	0.00	NaN	NaN
Biclique c:6, p:5	60	0	0.00	3	0.00	0.00	NaN
Biclique c:8, p:2	82	1	0.00	3	0.00	NaN	NaN
Biclique c:6, p:4	125	2	0.00	8	0.00	0.28	NaN
Biclique c:6, p:3	424	7	0.01	32	0.00	0.20	NaN
Biclique c:7, p:2	453	5	0.01	26	0.00	0.17	0.02
Biclique c:5, p:5	1,127	20	0.04	108	0.01	0.18	0.06
Biclique c:5, p:4	2,280	37	0.07	251	0.03	0.13	0.09
Biclique c:6, p:2	2,839	36	0.07	203	0.02	0.15	0.10
Biclique c:5, p:3	6,009	75	0.15	638	0.07	0.11	0.12
Biclique c:4, p:4	7,116	84	0.16	772	0.09	0.10	0.12
Biclique c:4, p:3	12,264	121	0.24	1,225	0.14	0.09	0.13
Biclique c:5, p:2	17,234	141	0.28	1,471	0.17	0.09	0.13
Biclique c:4, p:2	24,173	173	0.34	1,910	0.22	0.08	0.13
Biclique c:3, p:3	61,511	310	0.61	5,040	0.59	0.06	0.11
Biclique c:3, p:2	76,023	330	0.65	5,377	0.63	0.06	0.11
Biclique c:2, p:2	159,408	471	0.93	8,061	0.94	0.06	0.10

Supplementary Table 7. *In relation to Figure 6c. Statistical results of biclique calculations for human with different minimum number of compounds (c) and proteins (p) on each side of the biclique using a confidence threshold of 0.4 and allowing insertion of one edge. The number of edges represents the number of predicted interactions. Datasets are sorted according to the number of edges. Dataset with maximum F1 score referred to in the text is highlighted. Note that the underlying reference data was unbalanced with mean number of positives n= 2,099 and the mean number of negatives n = 11,458.*

Dataset	# Edges	# TP	TPR	# FP	FPR	PPV	F1
Biclique c:9, p:8	1,648	651	0.31	6	0.00	0.99	0.47
Biclique c:8, p:9	1,928	658	0.31	7	0.00	0.99	0.48
Biclique c:9, p:6	2,447	752	0.36	8	0.00	0.99	0.53
Biclique c:9, p:5	2,990	800	0.38	10	0.00	0.99	0.55
Biclique c:8, p:5	3,904	857	0.41	13	0.00	0.99	0.58
Biclique c:8, p:4	5,225	906	0.43	15	0.00	0.98	0.60
Biclique c:8, p:3	7,257	980	0.47	21	0.00	0.98	0.63
Biclique c:7, p:4	8,442	993	0.47	28	0.00	0.97	0.64
Biclique c:7, p:3	11,234	1,073	0.51	39	0.00	0.97	0.67
Biclique c:8, p:2	13,115	1,077	0.51	44	0.00	0.96	0.67
Biclique c:6, p:6	13,667	991	0.47	42	0.00	0.96	0.63
Biclique c:6, p:5	15,348	1,044	0.50	47	0.00	0.96	0.65
Biclique c:6, p:4	17,936	1,100	0.52	52	0.00	0.95	0.68
Biclique c:7, p:2	19,160	1,170	0.56	68	0.01	0.95	0.70
Biclique c:6, p:3	22,291	1,177	0.56	70	0.01	0.94	0.70
Biclique c:6, p:2	34,180	1,282	0.61	114	0.01	0.92	0.73
Biclique c:5, p:5	42,367	1,207	0.57	130	0.01	0.90	0.70
Biclique c:5, p:4	47,868	1,265	0.60	144	0.01	0.90	0.72
Biclique c:5, p:3	55,987	1,335	0.64	169	0.01	0.89	0.74
Biclique c:5, p:2	73,593	1,424	0.68	225	0.02	0.86	0.76
Biclique c:4, p:7	117,610	1,369	0.65	318	0.03	0.81	0.72
Biclique c:4, p:5	139,361	1,459	0.70	375	0.03	0.80	0.74
Biclique c:4, p:4	154,342	1,503	0.72	412	0.04	0.79	0.75
Biclique c:4, p:3	172,985	1,564	0.74	456	0.04	0.77	0.76
**Biclique c:4, p:2**	**201,212**	**1,638**	**0.78**	**534**	**0.05**	**0.75**	**0.77**
Biclique c:3, p:9	363,364	1,579	0.75	790	0.07	0.67	0.71

Biclique c:3, p:3	563,164	1,801	0.86	1,112	0.10	0.62	0.72
Biclique c:3, p:2	621,522	1,856	0.88	1,221	0.11	0.60	0.72
Biclique c:2, p:9	982,566	1,811	0.86	1,717	0.15	0.51	0.64
Biclique c:2, p:2	1,592,215	2,028	0.97	2,557	0.22	0.44	0.61

Supplementary Table 8. *In relation to Figure 6d. Statistical results of biclique calculations for human randomized networks with different minimum number of compounds (c) and proteins (p) on each side of the biclique using a confidence threshold of 0.4 and allowing insertion of one edge. The number of edges represents the number of predicted interactions. Datasets are sorted according to the number of edges. Dataset with maximum F1 score referred to in the text is highlighted. Note that the underlying reference data was unbalanced with mean number of positives n= 1,888 and the mean number of negatives n = 11,501.*

Dataset	# Edges	# TP	TPR	# FP	FPR	PPV	F1
Biclique c:9, p:8	31	0	0.00	1	0.00	NaN	NaN
Biclique c:9, p:6	95	1	0.00	1	0.00	NaN	NaN
Biclique c:8, p:9	153	1	0.00	2	0.00	NaN	NaN
Biclique c:9, p:5	274	2	0.00	3	0.00	NaN	NaN
Biclique c:8, p:5	1,628	13	0.01	14	0.00	0.46	0.01
Biclique c:8, p:4	4,061	25	0.01	27	0.00	0.46	0.03
Biclique c:7, p:4	14,262	74	0.04	69	0.01	0.51	0.07
Biclique c:8, p:3	19,309	76	0.04	83	0.01	0.47	0.07
Biclique c:6, p:6	23,600	129	0.07	108	0.01	0.54	0.12
Biclique c:6, p:5	33,118	159	0.08	140	0.01	0.53	0.14
Biclique c:7, p:3	39,876	147	0.08	148	0.01	0.49	0.13
Biclique c:6, p:4	47,983	201	0.11	182	0.02	0.53	0.18
Biclique c:6, p:3	87,188	298	0.16	291	0.03	0.51	0.24
Biclique c:5, p:5	117,156	395	0.21	393	0.03	0.50	0.29
Biclique c:5, p:4	154,501	455	0.24	482	0.04	0.49	0.32
Biclique c:8, p:2	206,770	383	0.20	533	0.05	0.42	0.27
Biclique c:5, p:3	218,890	554	0.29	639	0.06	0.46	0.36
Biclique c:4, p:7	258,861	664	0.35	754	0.07	0.47	0.40
Biclique c:7, p:2	303,101	507	0.27	742	0.06	0.41	0.32
Biclique c:4, p:5	380,442	779	0.41	1,010	0.09	0.44	0.42

Biclique c:6, p:2	426,662	649	0.34	1,021	0.09	0.39	0.36
Biclique c:4, p:4	472,403	845	0.45	1,201	0.10	0.41	0.43
Biclique c:4, p:3	592,246	921	0.49	1,438	0.13	0.39	0.43
Biclique c:5, p:2	604,874	838	0.44	1,433	0.12	0.37	0.40
Biclique c:3, p:9	667,629	1,046	0.55	1,512	0.13	0.41	0.47
Biclique c:4, p:2	983,616	1,088	0.58	2,190	0.19	0.33	0.42
Biclique c:3, p:3	1,628,943	1,348	0.71	3,130	0.27	0.30	0.42
Biclique c:2, p:9	1,849,606	1,478	0.78	3,178	0.28	0.32	0.45
Biclique c:3, p:2	2,027,395	1,423	0.75	3,797	0.33	0.27	0.40
Biclique c:2, p:2	4,392,612	1,725	0.91	6,587	0.57	0.21	0.34

Supplementary Table 9. *In relation to Supplementary Figure 4c. Statistical results of biclique calculations for human with different minimum number of compounds (c) and proteins (p) on each side of the biclique using a confidence threshold of 0.4 and allowing insertion of two edges. The number of edges represents the number of predicted interactions. Datasets are sorted according to the number of edges. Dataset with maximum F1 score referred to in the text is highlighted. Note that the underlying reference data was unbalanced with mean number of positives n= 2,099 and the mean number of negatives n = 11,458.*

Dataset	# Edges	# TP	TPR	# FP	FPR	PPV	F1
Biclique c:9, p:8	3,204	769	0.37	15	0.00	0.98	0.53
Biclique c:9, p:6	4,442	873	0.42	16	0.00	0.98	0.58
Biclique c:8, p:9	4,739	766	0.37	23	0.00	0.97	0.53
Biclique c:9, p:5	5,710	913	0.44	21	0.00	0.98	0.60
Biclique c:8, p:5	8,631	966	0.46	31	0.00	0.97	0.62
Biclique c:8, p:4	9,600	1,016	0.48	34	0.00	0.97	0.65
Biclique c:8, p:3	11,897	1,096	0.52	46	0.00	0.96	0.68
Biclique c:8, p:2	18,979	1,187	0.57	76	0.01	0.94	0.71
Biclique c:7, p:4	20,732	1,140	0.54	63	0.01	0.95	0.69
Biclique c:7, p:3	24,739	1,214	0.58	81	0.01	0.94	0.72
Biclique c:7, p:2	34,242	1,304	0.62	114	0.01	0.92	0.74
Biclique c:6, p:6	44,483	1,177	0.56	136	0.01	0.90	0.69
Biclique c:6, p:5	48,985	1,221	0.58	151	0.01	0.89	0.70
Biclique c:6, p:4	54,294	1,284	0.61	172	0.02	0.88	0.72

Biclique c:6, p:3	62,067	1,359	0.65	200	0.02	0.87	0.74
**Biclique c:6, p:2**	**80,384**	**1,443**	**0.69**	**249**	**0.02**	**0.85**	**0.76**
Biclique c:4, p:7	151,568	1,454	0.69	385	0.03	0.79	0.74
Biclique c:5, p:5	158,559	1,478	0.70	403	0.04	0.79	0.74
Biclique c:5, p:4	172,838	1,514	0.72	447	0.04	0.77	0.75
Biclique c:4, p:5	184,450	1,536	0.73	470	0.04	0.77	0.75
Biclique c:5, p:3	202,275	1,581	0.75	498	0.04	0.76	0.76
Biclique c:4, p:4	204,738	1,570	0.75	505	0.04	0.76	0.75
Biclique c:4, p:3	233,901	1,628	0.78	567	0.05	0.74	0.76
Biclique c:5, p:2	242,027	1,646	0.78	601	0.05	0.73	0.76
Biclique c:4, p:2	278,556	1,687	0.80	686	0.06	0.71	0.75
Biclique c:3, p:9	379,548	1,631	0.78	831	0.07	0.66	0.72
Biclique c:3, p:3	596,552	1,833	0.87	1,175	0.10	0.61	0.72
Biclique c:3, p:2	668,174	1,885	0.90	1,299	0.11	0.59	0.71
Biclique c:2, p:9	1,009,623	1,854	0.88	1,733	0.15	0.52	0.65
Biclique c:2, p:2	1,631,265	2,046	0.98	2,613	0.23	0.44	0.61

Supplementary Table 10. *In relation to Supplementary Figure 4d. Statistical results of biclique calculations for human randomized networks with different minimum number of compounds (c) and proteins (p) on each side of the biclique using a confidence threshold of 0.4 and allowing insertion of one edge. The number of edges represents the number of predicted interactions. Datasets are sorted according to the number of edges. Dataset with maximum F1 score referred to in the text is highlighted. Note that the underlying reference data was unbalanced with mean number of positives n= 1,880 and the mean number of negatives n = 11,506.*

Dataset	# Edges	# TP	TPR	# FP	FPR	PPV	F1
Biclique c:9, p:8	284	4	0.00	4	0.00	0.43	0.00
Biclique c:9, p:6	749	7	0.00	8	0.00	NaN	NaN
Biclique c:8, p:9	1,038	13	0.01	9	0.00	0.63	0.01
Biclique c:9, p:5	1,969	12	0.01	15	0.00	0.51	0.01
Biclique c:8, p:5	9,152	55	0.03	50	0.00	0.52	0.06
Biclique c:8, p:4	16,345	88	0.05	75	0.01	0.54	0.09
Biclique c:8, p:3	37,299	156	0.08	140	0.01	0.52	0.14
Biclique c:7, p:4	54,119	241	0.13	200	0.02	0.55	0.21

Biclique c:6, p:6	80,633	353	0.19	296	0.03	0.54	0.28
Biclique c:7, p:3	97,031	342	0.18	320	0.03	0.52	0.27
Biclique c:6, p:5	114,132	428	0.23	389	0.03	0.52	0.32
Biclique c:6, p:4	166,065	520	0.28	517	0.04	0.50	0.36
Biclique c:8, p:2	245,524	452	0.24	620	0.05	0.42	0.31
Biclique c:6, p:3	257,527	636	0.34	734	0.06	0.46	0.39
Biclique c:4, p:7	300,641	761	0.41	850	0.07	0.47	0.44
Biclique c:5, p:5	355,552	814	0.43	977	0.08	0.45	0.44
Biclique c:7, p:2	373,966	628	0.33	918	0.08	0.41	0.37
Biclique c:4, p:5	464,224	904	0.48	1,200	0.10	0.43	0.45
Biclique c:5, p:4	485,839	918	0.49	1,242	0.11	0.42	0.45
Biclique c:4, p:4	600,488	990	0.53	1,470	0.13	0.40	0.46
Biclique c:6, p:2	601,977	865	0.46	1,429	0.12	0.38	0.41
Biclique c:5, p:3	683,556	1,032	0.55	1,630	0.14	0.39	0.45
Biclique c:3, p:9	741,742	1,139	0.61	1,654	0.14	0.41	0.49
Biclique c:4, p:3	795,442	1,079	0.57	1,839	0.16	0.37	0.45
Biclique c:5, p:2	1,112,779	1,181	0.63	2,436	0.21	0.33	0.43
Biclique c:4, p:2	1,254,992	1,227	0.65	2,694	0.23	0.31	0.42
Biclique c:3, p:3	1,792,491	1,434	0.76	3,408	0.30	0.30	0.43
Biclique c:2, p:9	2,014,916	1,539	0.82	3,415	0.30	0.31	0.45
Biclique c:3, p:2	2,245,826	1,519	0.81	4,146	0.36	0.27	0.40
Biclique c:2, p:2	4,740,224	1,776	0.94	6,998	0.61	0.20	0.33

Supplementary Table 11. *KEGG enrichment for the E. coli input network (threshold 0.4) with p.adjust <=0.01, ordered by p.adjust.*

Description	GeneRatio	BgRatio	p.adjust	fold.enrichment
Biosynthesis of secondary metabolites	287/1076	339/1706	2.58E-20	1.34
Biosynthesis of cofactors	126/1076	142/1706	3.35E-11	1.41
Biosynthesis of amino acids	106/1076	117/1706	7.58E-11	1.44
Microbial metabolism in diverse environments	215/1076	267/1706	3.70E-10	1.28
Carbon metabolism	95/1076	110/1706	3.39E-07	1.37
Alanine, aspartate and glutamate metabolism	33/1076	33/1706	2.45E-06	1.59
Pyrimidine metabolism	48/1076	51/1706	2.45E-06	1.49
Pyruvate metabolism	53/1076	59/1706	2.56E-05	1.42
Glycolysis / Gluconeogenesis	43/1076	47/1706	6.99E-05	1.45
Phenylalanine, tyrosine and tryptophan biosynthesis	21/1076	21/1706	4.34E-04	1.59
O-Antigen nucleotide sugar biosynthesis	21/1076	21/1706	4.34E-04	1.59
Folate biosynthesis	25/1076	26/1706	6.32E-04	1.52
Butanoate metabolism	32/1076	35/1706	8.47E-04	1.45
Arginine biosynthesis	18/1076	18/1706	1.39E-03	1.59
Fatty acid degradation	15/1076	15/1706	5.24E-03	1.59
Citrate cycle (TCA cycle)	26/1076	29/1706	6.59E-03	1.42
Propanoate metabolism	32/1076	37/1706	6.59E-03	1.37
beta-Alanine metabolism	14/1076	14/1706	6.59E-03	1.59
Biotin metabolism	14/1076	14/1706	6.59E-03	1.59
Pentose phosphate pathway	28/1076	32/1706	8.08E-03	1.39
Lysine biosynthesis	13/1076	13/1706	9.06E-03	1.59
One carbon pool by folate	13/1076	13/1706	9.06E-03	1.59
Purine metabolism	61/1076	78/1706	9.06E-03	1.24

Supplementary Table 12. *KEGG enrichment for the E. coli Biclique proteins c:5/p:2, p.adjust <= 0.01, n = 463 input proteins (threshold 0.4) with p.adjust <=0.01, ordered by p.adjust.*

Description	GeneRatio	BgRatio	p.adjust	fold.enrichment
Ribosome	36/299	78/1706	1.38E-07	2.63
Pyrimidine metabolism	22/299	51/1706	4.95E-04	2.46
Citrate cycle (TCA cycle)	15/299	29/1706	6.11E-04	2.95
2-Oxocarboxylic acid metabolism	13/299	26/1706	2.60E-03	2.85
Microbial metabolism in diverse environments	67/299	267/1706	6.54E-03	1.43
Pyruvate metabolism	21/299	59/1706	7.07E-03	2.03
Alanine, aspartate and glutamate metabolism	14/299	33/1706	7.07E-03	2.42

Supplementary Table 13. *KEGG enrichment for the E. coli Predicted proteins c:5/p:2, p.adjust <= 0.01, n = 420 input proteins (threshold 0.4) with p.adjust <=0.01, ordered by p.adjust.*

Description	GeneRatio	BgRatio	p.adjust	fold.enrichment
Ribosome	36/267	78/1706	4.35E-09	2.95
Citrate cycle (TCA cycle)	14/267	29/1706	1.21E-03	3.08
Butanoate metabolism	14/267	35/1706	9.84E-03	2.56

Supplementary Table 14*. Metabolites considered "currency metabolites" contained in the E. coli network.*

KEGG ID	Chemical ID	Abbreviation
C00002	CIDm00000238	ATP
C00003	CIDm00000925	NAD+
C00004	CIDm00000928	NADH
C00005	CIDm00000930	NADPH
C00006	CIDm00000929	NADP+
C00008	CIDm00000197	ADP
C00010	CIDm00000317	CoA
C00013	CIDm00001023	Pyrophosphate
C00015	CIDm00001158	UDP
C00016	CIDm00000703	FAD
C00018	CIDm00001051	Pyridoxalphosphate
C00019	CIDm00001079	S-Adenosyl-L-methionine
C00020	CIDm00000224	AMP
C00021	CIDm00000193	S-Adenosylhomocysteine
C00035	CIDm00000730	GDP
C00044	CIDm00000762	GTP
C00055	CIDm00000314	CMP
C00061	CIDm00000710	FMN
C00063	CIDm00000593	CTP
C00075	CIDm00001181	UTP
C00105	CIDm00001172	UMP
C00112	CIDm00000290	CDP
C00113	CIDm00001024	PQQ
C00120	CIDm00000253	Biotin
C00144	CIDm00000761	GMP
C00175	CIDm00002891	Cobalt
C00194	CIDm00002891	Cobamidecoenzyme
C01352	CIDm00000705	FADH2

Supplementary Table 15. *In relation to Supplementary Figure 3. Statistical results of biclique calculations for E. coli with different minimum number of compounds (c) and proteins (p) on each side of the biclique using a confidence threshold of 0.4 and allowing insertion of one edge to complete a biclique. The number of edges represents the number of predicted interactions. Datasets are sorted according to the number of edges. All values are the mean values of n=10 repetitions. Note that the underlying reference data was unbalanced with a mean number of positives n= 403 and a mean number of negatives n = 4,332.*

Dataset	# Edges	# TP	TPR	# FP	FPR	PPV	F1
Biclique c:8, p:4	32	27	0.07	1	0.00	0.97	0.13
Biclique c:8, p:3	40	31	0.08	2	0.00	0.95	0.14
Biclique c:6, p:6	44	28	0.07	2	0.00	0.93	0.13
Biclique c:7, p:4	47	33	0.08	2	0.00	0.94	0.15
Biclique c:8, p:2	52	38	0.10	2	0.00	0.95	0.17
Biclique c:7, p:3	59	41	0.10	3	0.00	0.94	0.18
Biclique c:6, p:5	63	35	0.09	4	0.00	0.90	0.16
Biclique c:6, p:4	86	45	0.11	5	0.00	0.89	0.20
Biclique c:7, p:2	100	54	0.13	6	0.00	0.91	0.23
Biclique c:6, p:3	118	58	0.14	7	0.00	0.89	0.25
Biclique c:6, p:2	187	75	0.19	10	0.00	0.88	0.31
Biclique c:5, p:5	188	54	0.13	16	0.00	0.77	0.23
Biclique c:5, p:4	245	67	0.17	19	0.00	0.78	0.27
Biclique c:5, p:3	324	89	0.22	25	0.01	0.78	0.34
Biclique c:5, p:2	457	110	0.27	32	0.01	0.77	0.40
Biclique c:4, p:4	1,739	123	0.30	173	0.04	0.42	0.35
Biclique c:4, p:3	2,138	145	0.36	208	0.05	0.41	0.38
Biclique c:4, p:2	2,791	170	0.42	245	0.06	0.41	0.42
Biclique c:3, p:3	20,056	253	0.63	1,668	0.38	0.13	0.22
Biclique c:3, p:2	23,184	269	0.67	1,801	0.42	0.13	0.22
Biclique c:2, p:2	92,731	386	0.96	3,780	0.87	0.09	0.17

Supplementary Table 16. *In relation to Supplementary Figure 6. Statistical results of biclique calculations for the human network based on BindingDB, with different minimum number of compounds (c) and proteins (p) on each side of the biclique using a confidence threshold of 0.4 and allowing insertion of one edge. The number of edges represents the number of predicted interactions. Datasets are sorted according to the number of edges. Dataset with maximum F1 score referred to in the text is highlighted. Note that the underlying reference data was unbalanced with the number of positives n= 1,671 and the number of negatives n = 15,096.*

Dataset	# Edges	# TP	TPR	# FP	FPR	PPV	F1
Biclique c:9, p:8	1,100	455	0.27	6	0.00	0.99	0.43
Biclique c:8, p:9	1,273	463	0.28	7	0.00	0.99	0.43
Biclique c:9, p:6	1,799	564	0.34	7	0.00	0.99	0.50
Biclique c:9, p:5	2,272	608	0.36	7	0.00	0.99	0.53
Biclique c:8, p:5	2,914	644	0.39	11	0.00	0.98	0.55
Biclique c:8, p:4	3,954	698	0.42	13	0.00	0.98	0.59
Biclique c:8, p:3	5,718	771	0.46	17	0.00	0.98	0.63
Biclique c:7, p:4	6,154	768	0.46	24	0.00	0.97	0.62
Biclique c:7, p:3	8,230	840	0.50	31	0.00	0.96	0.66
Biclique c:6, p:6	8,652	748	0.45	39	0.00	0.95	0.61
Biclique c:6, p:5	10,332	798	0.48	44	0.00	0.95	0.64
Biclique c:8, p:2	11,501	863	0.52	44	0.00	0.95	0.67
Biclique c:6, p:4	12,364	849	0.51	48	0.00	0.95	0.66
Biclique c:7, p:2	15,473	926	0.55	68	0.00	0.93	0.70
Biclique c:6, p:3	15,610	917	0.55	58	0.00	0.94	0.69
Biclique c:6, p:2	26,584	1,005	0.60	111	0.01	0.90	0.72
Biclique c:5, p:5	29,527	928	0.56	109	0.01	0.89	0.69
Biclique c:5, p:4	33,798	971	0.58	122	0.01	0.89	0.70
Biclique c:5, p:3	40,913	1,044	0.62	152	0.01	0.87	0.73
**Biclique c:5, p:2**	**57,410**	**1,127**	**0.67**	**210**	**0.01**	**0.84**	**0.75**
Biclique c:4, p:7	81,368	1,029	0.62	275	0.02	0.79	0.69
Biclique c:4, p:5	100,901	1,122	0.67	332	0.02	0.77	0.72
Biclique c:4, p:4	112,593	1,166	0.70	374	0.02	0.76	0.73
Biclique c:4, p:3	130,513	1,222	0.73	424	0.03	0.74	0.74
**Biclique c:4, p:2**	**155,983**	**1,292**	**0.77**	**501**	**0.03**	**0.72**	**0.75**
Biclique c:3, p:9	260,600	1,235	0.74	683	0.05	0.64	0.69

Biclique c:3, p:3	429,800	1,448	0.87	1,047	0.07	0.58	0.70
Biclique c:3, p:2	479,654	1,497	0.90	1,164	0.08	0.56	0.69
Biclique c:2, p:9	849,723	1,418	0.85	1,705	0.11	0.45	0.59
Biclique c:2, p:2	1,423,686	1,618	0.97	2,544	0.17	0.39	0.55

Supplementary Table 17. *DeppConv-DTI prediction results, using BiPredict predicted interactions as input test dataset after training the model.*

Sample (n = 100,000)	# predicted interactions	# predicted non-interactions
1	77,469	22,531
2	77,616	22,384
3	77,568	22,384
4	77,505	22,495
5	77,839	22,161
**mean**	**77,599**	**22,397**

Supplementary Table 18. *In relation to Figure 8a. Statistical results of biclique calculations for E. coli with different minimum number of compounds (c) and proteins (p) on each side of the biclique using the KEGG network and allowing insertion of one edge to complete a biclique. The number of edges represents the number of predicted interactions. Datasets are sorted according to the number of edges. All values are the mean values of n=10 repetitions. Note that the underlying reference data was unbalanced with a mean number of positives n= 129 and a mean number of negatives n = 8,884. Dataset with maximum F1 score referred to in the text is highlighted.*

Dataset	# Edges	# TP	TPR	# FP	FPR	PPV	F1
Biclique c:4, p:7	3	2	0.02	0	0.0000	1.00	0.03
Biclique c:3, p:9	6	3	0.02	0	0.0000	0.90	0.04
Biclique c:9, p:5	14	0	0.00	0	0.0000	NA	NA
Biclique c:8, p:4	15	8	0.06	0	0.0000	1.00	0.11
Biclique c:8, p:5	16	1	0.01	0	0.0000	1.00	0.02
Biclique c:6, p:5	26	3	0.02	0	0.0000	NA	NA
Biclique c:7, p:4	34	10	0.07	0	0.0000	1.00	0.14
Biclique c:5, p:5	37	4	0.03	0	0.0000	1.00	0.06
Biclique c:6, p:4	58	12	0.10	0	0.0000	1.00	0.17
Biclique c:4, p:5	70	9	0.07	0	0.0000	0.95	0.13
Biclique c:5, p:4	75	15	0.12	0	0.0000	1.00	0.21

Biclique c:2, p:9	80	5	0.04	5	0.0006	0.51	0.07
Biclique c:8, p:3	94	15	0.12	0	0.0000	0.98	0.21
Biclique c:7, p:3	111	17	0.13	1	0.0001	0.96	0.23
Biclique c:8, p:2	121	20	0.16	1	0.0001	0.97	0.27
Biclique c:4, p:4	144	17	0.13	1	0.0001	0.95	0.23
Biclique c:6, p:3	147	16	0.13	1	0.0001	0.96	0.22
Biclique c:7, p:2	164	22	0.17	1	0.0001	0.94	0.28
Biclique c:5, p:3	166	19	0.15	1	0.0001	0.95	0.25
Biclique c:6, p:2	236	25	0.20	2	0.0002	0.93	0.32
Biclique c:5, p:2	265	29	0.22	2	0.0002	0.94	0.36
Biclique c:4, p:3	308	27	0.21	3	0.0003	0.91	0.34
Biclique c:4, p:2	447	38	0.30	4	0.0005	0.90	0.45
Biclique c:3, p:3	462	35	0.27	7	0.0008	0.83	0.40
**Biclique c:3, p:2**	**706**	**51**	**0.40**	**12**	**0.0013**	**0.81**	**0.53**
Biclique c:2, p:2	3,294	76	0.59	153	0.0172	0.33	0.43

Supplementary Table 19. *In relation to Figure 8b. Statistical results of biclique calculations for E. coli randomized network with different minimum number of compounds (c) and proteins (p) on each side of the biclique using the KEGG network and allowing insertion of one edge. The number of edges represents the number of predicted interactions. Datasets are sorted according to the number of edges. All values are the mean values of n=10 repetitions. Note that the underlying reference data was unbalanced with a mean number of positives n= 122 and a mean number of negatives n = 8,768.*

Dataset	# Edges	# TP	TPR	# FP	FPR	PPV	F1
Biclique c:3, p:3	2	0	0.00	0	0.0000	NA	NA
Biclique c:7, p:2	55	1	0.01	1	0.0001	0.50	0.02
Biclique c:5, p:2	87	0	0.00	2	0.0002	NA	NA
Biclique c:4, p:2	291	0	0.00	8	0.0009	0.03	NA
Biclique c:3, p:2	1,083	1	0.01	30	0.0035	0.05	NA
Biclique c:2, p:2	7,663	5	0.04	354	0.0403	0.02	0.02

Supplementary Table 20. *In relation to Figure 8c. Statistical results of biclique calculations for human with different minimum number of compounds (c) and proteins (p) on each side of the biclique using the KEGG network and allowing insertion of one edge. The number of edges represents the number of predicted interactions. Datasets are sorted according to the number of edges. Dataset with maximum F1 score referred to in the text is highlighted. Note that the underlying reference data was unbalanced with mean number of positives n= 373 and the mean number of negatives n = 4,080.*

Dataset	# Edges	# TP	TPR	# FP	FPR	PPV	F1
Biclique c:8, p:9	93	79	0.21	0	0.0001	1.00	0.35
Biclique c:9, p:8	105	84	0.22	0	0.0001	1.00	0.37
Biclique c:9, p:6	140	97	0.26	1	0.0002	0.99	0.41
Biclique c:9, p:5	146	103	0.28	0	0.0001	1.00	0.43
Biclique c:8, p:5	185	108	0.29	2	0.0004	0.99	0.45
Biclique c:6, p:6	200	100	0.27	2	0.0004	0.98	0.42
Biclique c:6, p:5	224	114	0.31	2	0.0004	0.98	0.47
Biclique c:8, p:4	236	114	0.31	2	0.0004	0.99	0.47
Biclique c:7, p:4	280	119	0.32	2	0.0004	0.99	0.48
Biclique c:5, p:5	290	123	0.33	3	0.0007	0.98	0.49
Biclique c:4, p:7	344	119	0.32	3	0.0007	0.98	0.48
Biclique c:6, p:4	354	131	0.35	2	0.0005	0.98	0.52
Biclique c:8, p:3	360	140	0.38	2	0.0004	0.99	0.54
Biclique c:5, p:4	465	143	0.38	3	0.0008	0.98	0.55
Biclique c:4, p:5	470	151	0.40	4	0.0009	0.98	0.57
Biclique c:7, p:3	492	148	0.40	2	0.0004	0.99	0.57
Biclique c:6, p:3	708	163	0.44	3	0.0006	0.98	0.60
Biclique c:4, p:4	742	174	0.47	4	0.0010	0.98	0.63
Biclique c:5, p:3	917	175	0.47	4	0.0009	0.98	0.63
Biclique c:8, p:2	1,170	174	0.47	2	0.0005	0.99	0.63
Biclique c:4, p:3	1,274	210	0.56	4	0.0011	0.98	0.71
Biclique c:7, p:2	1,376	180	0.48	2	0.0005	0.99	0.65
Biclique c:6, p:2	1,631	195	0.52	4	0.0009	0.98	0.68
Biclique c:5, p:2	1,835	207	0.56	6	0.0014	0.97	0.71
Biclique c:3, p:3	2,012	230	0.61	8	0.0020	0.97	0.75
Biclique c:4, p:2	2,363	242	0.65	7	0.0018	0.97	0.78

Biclique c:3, p:2	3,610	266	0.71	12	0.0030	0.96	0.82
**Biclique c:2, p:2**	**9,133**	**312**	**0.84**	**42**	**0.0102**	**0.88**	**0.86**

Supplementary Table 21. *In relation to Figure 8d. Statistical results of biclique calculations for human randomized networks with different minimum number of compounds (c) and proteins (p) on each side of the biclique using the KEGG network and allowing insertion of one edge. The number of edges represents the number of predicted interactions. Datasets are sorted according to the number of edges. Note that the underlying reference data was unbalanced with mean number of positives n= 291 and the mean number of negatives n = 3,813.*

Dataset	# Edges	# TP	TPR	# FP	FPR	PPV	F1
Biclique c:4, p:3	4	0	0.00	0	0.0000	NA	NA
Biclique c:2, p:9	62	0	0.00	0	0.0000	NA	NA
Biclique c:3, p:3	63	0	0.00	0	0.0001	NA	NA
Biclique c:8, p:2	70	0	0.00	0	0.0000	NA	NA
Biclique c:7, p:2	74	0	0.00	0	0.0001	NA	NA
Biclique c:6, p:2	302	1	0.00	2	0.0004	NA	NA
Biclique c:5, p:2	1,440	3	0.01	7	0.0017	0.35	NA
Biclique c:4, p:2	6,399	16	0.06	28	0.0073	0.36	0.10
Biclique c:3, p:2	23,462	51	0.17	95	0.0247	0.35	0.23
Biclique c:2, p:2	92,279	107	0.37	396	0.1035	0.21	0.27
